# Leveraging Hypoxia-Activated Prodrugs to Prevent Drug Resistance in Solid Tumors

**DOI:** 10.1371/journal.pcbi.1005077

**Published:** 2016-08-25

**Authors:** Danika Lindsay, Colleen M. Garvey, Shannon M. Mumenthaler, Jasmine Foo

**Affiliations:** 1 School of Mathematics, University of Minnesota, Minneapolis, Minnesota, United States of America; 2 Lawrence J. Ellison Institute for Transformative Medicine, University of Southern California, Los Angeles, California, United States of America; University of California Irvine, UNITED STATES

## Abstract

Experimental studies have shown that one key factor in driving the emergence of drug resistance in solid tumors is tumor hypoxia, which leads to the formation of localized environmental niches where drug-resistant cell populations can evolve and survive. Hypoxia-activated prodrugs (HAPs) are compounds designed to penetrate to hypoxic regions of a tumor and release cytotoxic or cytostatic agents; several of these HAPs are currently in clinical trial. However, preliminary results have not shown a survival benefit in several of these trials. We hypothesize that the efficacy of treatments involving these prodrugs depends heavily on identifying the correct treatment schedule, and that mathematical modeling can be used to help design potential therapeutic strategies combining HAPs with standard therapies to achieve long-term tumor control or eradication. We develop this framework in the specific context of EGFR-driven non-small cell lung cancer, which is commonly treated with the tyrosine kinase inhibitor erlotinib. We develop a stochastic mathematical model, parametrized using clinical and experimental data, to explore a spectrum of treatment regimens combining a HAP, evofosfamide, with erlotinib. We design combination toxicity constraint models and optimize treatment strategies over the space of tolerated schedules to identify specific combination schedules that lead to optimal tumor control. We find that (i) combining these therapies delays resistance longer than any monotherapy schedule with either evofosfamide or erlotinib alone, (ii) sequentially alternating single doses of each drug leads to minimal tumor burden and maximal reduction in probability of developing resistance, and (iii) strategies minimizing the length of time after an evofosfamide dose and before erlotinib confer further benefits in reduction of tumor burden. These results provide insights into how hypoxia-activated prodrugs may be used to enhance therapeutic effectiveness in the clinic.

## Author Summary

It has been suggested that one key factor driving the emergence of drug resistance is the spatial heterogeneity in the distribution of drug and oxygen throughout a tumor due to disorganized tumor vasculatures. Researchers have developed a class of novel drugs that penetrate to hypoxic regions where they are activated to kill tumor cells. The inclusion of these drugs, called hypoxia-activated prodrugs (HAPs) alongside standard therapies in combination may be the key to long-term tumor control or eradication. However, identifying the right timing and administration sequence of combination therapies is an extremely difficult task, and the time and human costs of clinical trials to investigate even a few options is often prohibitive. In this work we design a mathematical model based upon evolutionary principles to investigate the potential of combining HAPs with standard targeted therapy for a specific example in non-small cell lung cancer. We formulate novel toxicity constraints from existing clinical data to estimate the shape of the tolerated drug combination treatment space. We find that (i) combining these therapies delays resistance longer than any monotherapy schedule with either evofosfamide or erlotinib alone, and (ii) the best strategy for combination involves single doses of each drug sequentially administered in an alternating sequence. These model predictions of tumor dynamics during treatment provide insight into the role of the tumor microenvironment in combination therapy and identify treatment hypotheses for further experimental and clinical testing.

## Introduction

Solid tumor vasculature is characterized by a disorganized, aberrant network structure of tortuous, hyperpermeable blood vessels [1]. These characteristics lead to nonuniform spatial distributions of drug and oxygen (as well as other nutrients and growth factors) throughout tumors, which in turn have been implicated in the emergence and evolution of resistance [2–7]. Indeed, several recent studies have demonstrated that the presence of spatial gradients of drug in an environment can accelerate the emergence of antibiotic resistance in bacteria [8, 9]. One explanation for this phenomenon is that regions of low drug concentration generate local niches where sustained cell proliferation drives the production of new genetic variants. These spatial regions often coincide with hypoxic (low oxygen) conditions where drug-resistant variants may possess a survival advantage over drug-sensitive cells [2, 10–13], thus enabling the establishment of stable pockets of drug resistance in tumor regions not easily accessible by drugs. In light of these observations, one strategy proposed is to design therapy regimens that exploit the interaction between tumor cell populations and their environments to achieve long-term tumor eradication or control.

Hypoxia is defined as reduced levels of molecular oxygen (typically less than 1%) in tissue. In contrast, ambient air exists at approximately 21% and most human organs have oxygen levels in the range of 2% to 9% [14]. The prevalence of hypoxic regions in solid tumors has led to the development of hypoxia-activated prodrugs (HAPs), which are compounds designed to metabolize into active drugs upon entry into hypoxic environments [15–18]. For example, one such compound, evofosfamide, consists of a radical anion linked to a potent DNA-alkylating agent which penetrates effectively through tissues under normoxic (normal oxygen) conditions. Under hypoxic conditions, however, the radical anion undergoes irreversible fragmentation and releases the activated drug into the tumor [18, 19]. This type of novel action allows evofosfamide to penetrate and target cancer cells within hypoxic region of a tumor, unlike standard therapies whose range is often confined to well-vascularized, normoxic regions.

Currently, several HAPs are in clinical trials [20]. Tirapazamine was the first HAP to be tested in the clinic, in combination with both chemotherapy and radiotherapy; however, results did not show any significant therapeutic benefit. It was thought that off-target toxicity and insufficient tissue penetration were the primary contributing factors to this result. More recently, evofosfamide, which has a superior tissue-penetration ability, underwent Phase III testing in combination with chemotherapies for pancreatic cancer and soft tissue sarcoma. Neither of these trials demonstrated a significant survival benefit. However, given the response kinetics of hypoxic cancer cells to these therapies in preclinical studies, we hypothesize that the potential of HAPs has not yet been fully realized in previous clinical trials, and that mathematical modeling may be beneficial in identifying the combination treatment strategies that lead to survival benefit. We have demonstrated in previous work that identifying the right dosing schedule is important in improving cancer treatment outcomes, and further that mathematical modeling is an effective tool to help identify optimal administration strategies [21–23]. In particular, we demonstrated in [23] that altering the timing of treatment periods in sequential combination therapies may prevent or delay the emergence of drug resistance.

This work builds upon a large body of literature on evolutionary modeling of drug resistance in cancer (see, e.g. the review [24] and references therein). Here we discuss a few recent contributions to modeling combination therapies in cancer. For example, Komarova et. al. [25] utilized a stochastic birth-death process model to study the impact of combination therapies in Chronic Myeloid Leukemia, finding that a combination of two but not three drugs should be used in the prevention of drug resistance. In a later work by the same authors, Katouli et. al. [26] designed a general algorithm to compare combination treatment protocols in Chronic Myeloid Leukemia according to their cross-resistance properties, and to identify the protocols with the highest probability of treatment success. Most recently, Bozic et al. [27] developed a mathematical model that predicts responses to combinations of targeted inhibitors in melanoma patients. Using this model, the author predicted that combinations of two or three drugs will be far more effective than sequential treatment with the same agents, with the potential for complete cure.

Here we will focus our modeling efforts on designing HAP-targeted combination treatment strategies for non-small cell lung cancer (NSCLC). Erlotinib is a tyrosine kinase inhibitor commonly used to treat EGFR-mutant non-small cell lung cancer [28–30]. However, most patients develop resistance and disease progression within 12–18 months of starting treatment [31]. Consequently, novel approaches to prevent, or at least delay, the onset of resistance to erlotinib are of great clinical importance. A significant amount of research has been dedicated to improving treatment of non-small cell lung cancer. Several studies have shown that it may be beneficial to continue therapy with tyrosine kinase inhibitors such as erlotinib even after the point of disease progression [32–34]. Previous work has focused on the use of mathematical models of tumor growth and resistance during erlotinib treatment to optimize therapeutic strategies and minimize a patient’s risk of resistance [21, 22]. Another approach that has been extensively studied is the use of combination therapy to mitigate resistance to erlotinib [23, 35, 36]. However, these studies lack a consideration for the heterogeneous oxygen and drug distributions throughout the tumor, and in particular their role in mediating tumor response to therapy and the emergence of drug resistance. Recently, we have demonstrated through modeling efforts that the consideration of heterogeneous tumor oxygenation and drug concentration reveals dramatically different treatment outcomes and evolutionary responses to therapy when compared to models under homogeneous environmental conditions [2].

In this work, we investigate the potential benefits of using a HAP in combination with standard therapy to prevent the emergence of resistance to erlotinib in non-small cell lung cancer. We design a stochastic mathematical model, with parameters informed by experimental data, to describe the evolutionary dynamics of a cancer cell population within a heterogeneous tumor microenvironment during treatment with erlotinib and evofosfamide. Using this model, we show that a combination treatment strategy results in treatment outcomes preferable to those resulting from monotherapy with either of these drugs alone. We also use a novel approach to define toxicity constraints for this combination therapy, which allows us to optimize treatment strategies over the space of tolerated dosing schedules using both drugs in order to minimize tumor burden and probability of resistance. Determining toxicity constraint profiles for combination therapies could have significant clinical implications as severe toxicity issues is one of the major reasons that HAP combinations have not been successful in clinical trials.

## Methods

In the following we describe an evolutionary mathematical modeling approach to evaluate the impact of combination erlotinib-evofosfamide therapy on a NSCLC tumor cell population, in which each tumor cell response is dependent upon local environmental concentrations of oxygen and drug. The pseudo-spatial model is comprised of a weighted series of environmental compartments whose oxygen and drug profiles mirror tumor physiologic data. The model is parameterized using (i) experimentally calculated growth rates under a spectrum of environmental perturbations of oxygen and erlotinib concentration, (ii) published experimental results on cell viability in response to evofosfamide therapy, (iii) tumor oxygenation measurements, and (iv) pharmacokinetic data mapping evofosfamide and erlotinib dose to plasma concentration. We also utilize clinical trial data for both therapies to design a methodology for inferring toxicity constraints on the space of possible combination treatment strategies.

### Evolutionary Model

**Fig 1:**
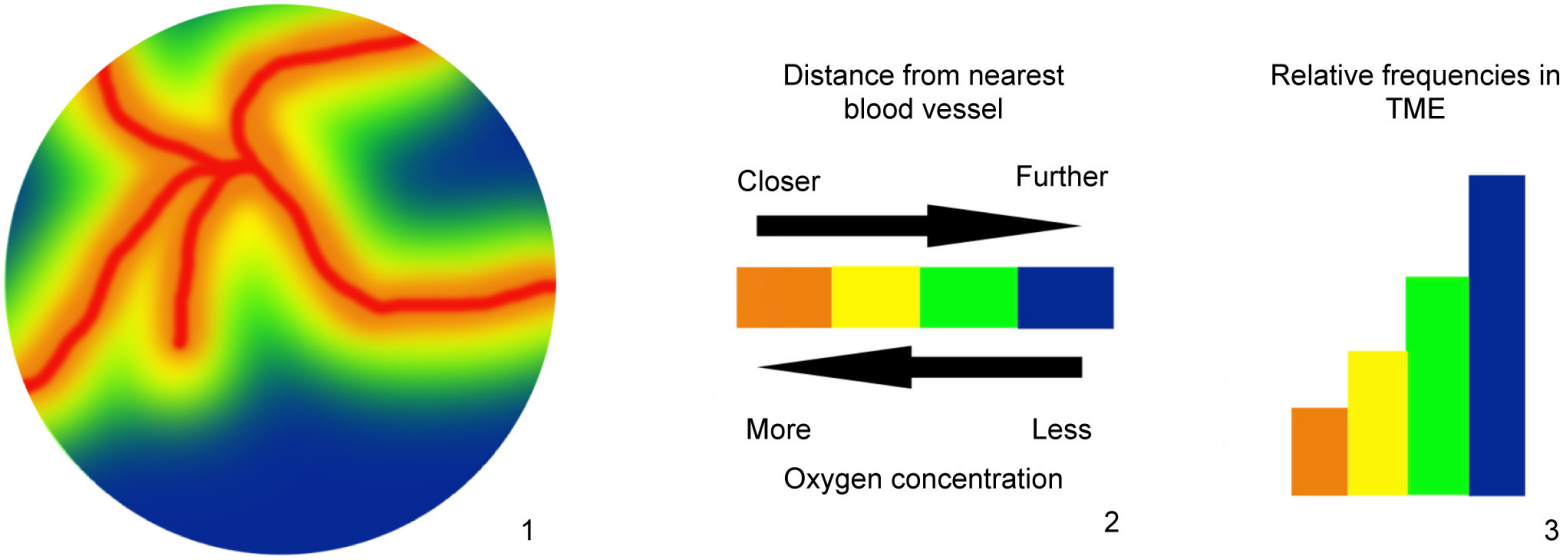
Tumor microenvironment modeling process. This schematic shows the process used to model the tumor microenvironment as a set of discrete compartments. A series of compartments is defined based on various distances from the nearest blood vessel, and the oxygen concentration in each compartment is calculated accordingly. The relative weights of the compartments are determined based on experimental observations of oxygen partial pressure distribution in solid tumors.

In order to model tumor evolution within an environment with heterogeneous oxygen and drug concentrations, we consider a stochastic population dynamic process in which the cell population is distributed amongst a series of habitats with varying oxygen and drug profiles. Oxygen concentration within the tumor decays exponentially as a function of distance from the nearest blood vessel. This decay rate is parameterized in the model based on estimates of the half-length away from the blood vessel [2]. This is used to define the oxygen concentration in each microenvironmental compartment; hence every compartment corresponds to a volume some distance from the nearest blood vessel. To estimate the relative contributions of each of these compartments to the tumor microenvironment, we utilize experimental data capturing relative frequencies of a spectrum of oxygen partial pressures throughout solid tumors [37]. We consider a total of 32 environmental compartments in which compartment *i* has an oxygen partial pressure of 2.5 ⋅ *i* mmHg to mirror this data. We then construct a mixture model of compartments in which the weighting of each compartment is determined based on the relative frequency of its corresponding oxygen partial pressure in the experimental profile. A schematic of this process is depicted in Fig 1.

Within each compartment, we use a multi-type, non-homogeneous, continuous-time birth-death process to model the population of cancer cells during treatment. We assume for now that the evolutionary dynamics within each microenvironmental compartment are independent. (This assumption will be relaxed later, see section on Migration in S1 Text.) The number of erlotinib-sensitive cells in compartment *i* at time *t* is denoted by *X*_*i*_(*t*), and the number of erlotinib-resistant cells in compartment *i* at time *t* is given by *Y*_*i*_(*t*). The joint process **X**_*i*_(*t*) = (*X*_*i*_(*t*), *Y*_*i*_(*t*)) represents the combined state of the sensitive and resistant cell populations in compartment *i* at time *t*. In compartment *i*, erlotinib-sensitive cells proliferate and die with rates *λ*_*X*,*i*_(*t*) and *μ*_*X*,*i*_(*t*), respectively, while erlotinib-resistant cells proliferate and die with rates *λ*_*Y*,*i*_(*t*) and *μ*_*Y*,*i*_(*t*). These birth and death rates reflect the effect of treatment on the cancer cell population in an environmental compartment *i*, and therefore depend on the concentrations of oxygen and both drugs found in that compartment at time *t*. During every sensitive cell division, a mutation may arise with some small probability *u*, giving rise to a new resistant cell. This mutation rate falls in the range between 10^−8^ and 10^−6^ [21, 38, 39]. Here we use *u* = 10^−7^. We consider an initial population of *M* = 1.6 ⋅ 10^6^ erlotinib-sensitive cancer cells and zero resistant cells. The number of sensitive cells *M*_*i*_ initially in compartment *i* is calculated using the relative compartment weights.

The evolutionary dynamics within each microenvironmental compartment can be described using analytic approximations for the probability of resistance and means of the sensitive and resistant cell populations. The derivations of these analytic expressions are outlined elsewhere [40]. Because *u* is very small, we approximate 1 − *u* ≈ 1 in the following. Then the population of sensitive cells *X*_*i*_(*t*) in compartment *i* can be described using a simple birth-death process. Hence the mean number of sensitive cells at time *t* in this compartment is

$$E\left[X_{i}\left(t\right)\right]=M_{i}\exp\left[\int_{0}^{t}\left(\lambda_{X,i}\left(\tau \right)-\mu_{X,i}\left(\tau \right)\right)d\tau \right].$$ (1)

The mean number of resistant cells in this compartment at time *t* is

$$E\left[Y_{i}\left(t\right)\right]=\int_{0}^{t}b_{i}\left(\tau \right)\exp\left[\int_{0}^{t-\tau }\left(\lambda_{Y,i}\left(\tau +\eta \right)-\mu_{Y,i}\left(\tau +\eta \right)\right)d\eta \right]d\tau ,$$ (2)

where *b*_*i*_(*t*), the rate of production at time *t* of the resistant cells from the sensitive cell population in compartment *i*, is given by the formula

$$b_{i}\left(t\right)=M_{i}\exp\left[\int_{0}^{t}\left(\lambda_{X,i}\left(\tau \right)-\mu_{X,i}\left(\tau \right)\right)d\tau \right]\lambda_{X,i}\left(t\right)u.$$

Lastly, the probability of resistance in compartment *i* at time *t* is

$$P\left[Y_{i}\left(t\right)>0\right]=1-\exp\left[\int_{0}^{t}-b_{i}\left(T\right)\left(1-P_{i}^{ext}\left(T,t\right)\right)dT\right].$$ (3)

$P_{i}^{ext}\left(T,t\right)$ represents the probability that a group of resistant cells originating from a single resistant cell produced at time *T* in compartment *i* is completely extinct by time *t*, and is given by the formula

$$P_{i}^{ext}\left(T,t\right)=\frac{\int_{0}^{t-T}\mu_{Y,i}\left(\tau +T\right)\omega_{i}\left(\tau ,T\right)d\tau }{1+\int_{0}^{t-T}\mu_{Y,i}\left(\tau +T\right)\omega_{i}\left(\tau ,T\right)d\tau },$$

where

$$\omega_{i}\left(\tau ,T\right)=\exp\left[\int_{0}^{\tau }\left(\mu_{Y,i}\left(\eta +T\right)-\lambda_{Y,i}\left(\eta +T\right)\right)d\eta \right].$$

Now we calculate the probability of resistance and means of the sensitive and resistant cell populations in the entire tumor at time *t*. We can obtain the means at time *t* of the sensitive cell population *X*(*t*) and the resistant cell population *Y*(*t*) in the entire tumor by summing over all compartments:

$$\begin{array}{ll} E[X(t)] & =\sum_{i}E[X_{i}(t)],\\ E[Y(t)] & =\sum_{i}E[Y_{i}(t)]. \end{array}$$

Here, $E[X_{i}\left(t\right)]$ and $E[Y_{i}\left(t\right)]$ are given by Eqs (1) and (2), respectively. The mean tumor size at time *t* is given by

E[X(t)+Y(t)]=E[X(t)]+E[Y(t)].

The probability that there exists one or more resistant cells in compartment *i* at time *t* is $P[Y_{i}\left(t\right)>0]$. Then $1-P[Y_{i}\left(t\right)>0]$ is the probability of having zero resistant cells in this compartment at time *t*. Since we assume independence of the microenvironmental compartments, this implies the probability of having no resistant cells in the entire tumor at time *t* is given by $\prod_{i}(1-P[Y_{i}\left(t\right)>0])$. Therefore, the probability of resistance at time *t* is

$$P\left[Y\left(t\right)>0\right]=1-\prod_{i}\left(1-P[Y_{i}\left(t\right)>0]\right),$$

where $P[Y_{i}\left(t\right)>0]$ is given by Eq (3).

### Model Parameterization

The evolutionary dynamics of the tumor cell population depend on the birth and death rates of sensitive and resistant cells in each microenvironmental compartment. These rates, in turn, vary as the concentrations of both drugs change over time. To reflect this variation, we first define distinct functions describing the individual effects of erlotinib and evofosfamide on these birth and death rates. The growth kinetics of the cancer cell population during treatment by each of these drugs are estimated using a combination of pharmacokinetic and experimental cell viability data. In the following sections, functions pertaining to erlotinib are denoted with 1’s and functions pertaining to evofosfamide are denoted using 2’s. We note that the birth and death rates used in this parameterization are taken from *in vitro* data. Thus the specific time scale and cell population sizes of the model predictions are relevant to the *in vitro* setting.

#### Effect of erlotinib and oxygen on growth kinetics

**Fig 2:**
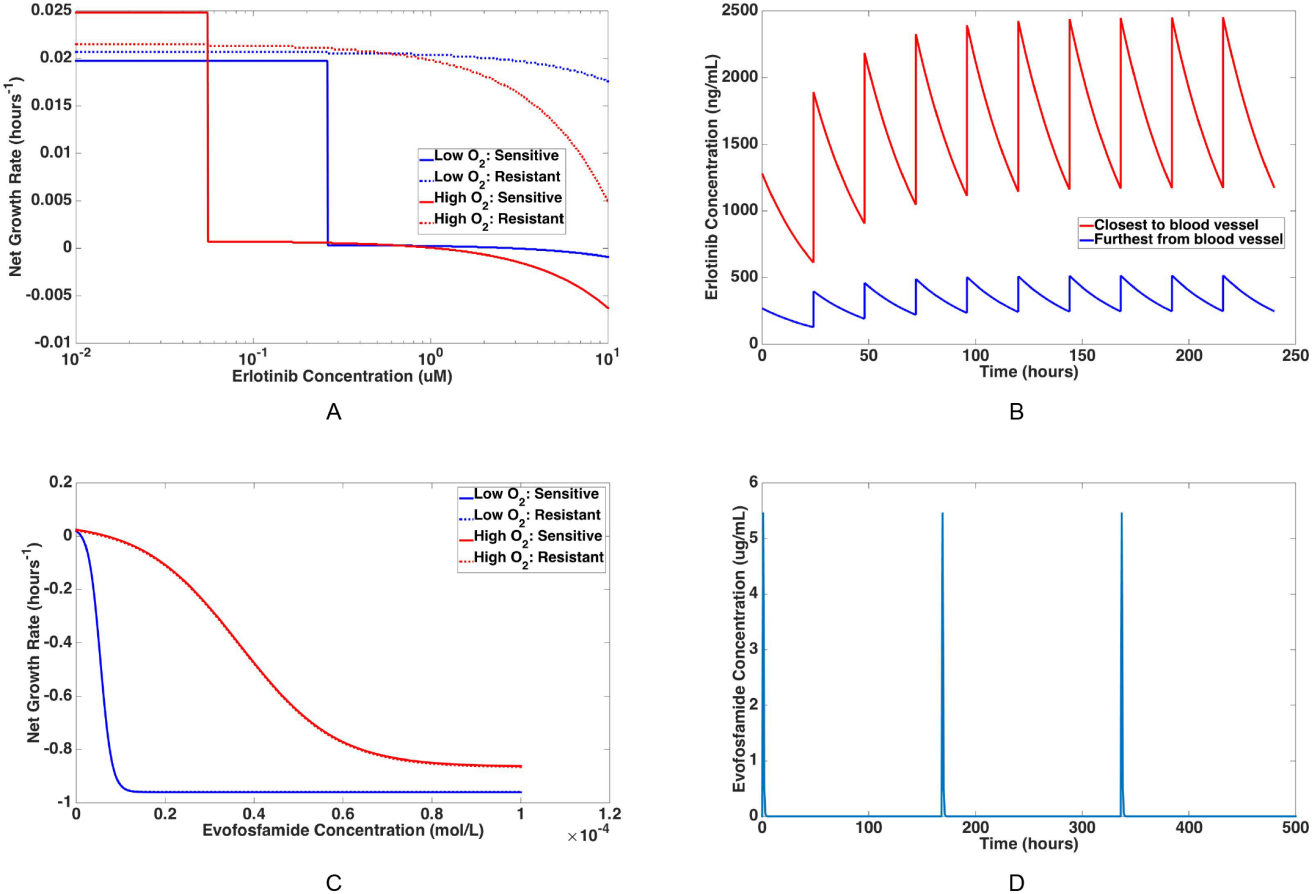
Net growth rate and plasma concentration functions for erlotinib and evofosfamide. Examples of net growth rates of sensitive and resistant cells are shown as functions of erlotinib concentration in (A) and evofosfamide concentration in (C). These rates are shown in blue for a low oxygen concentration (0.33%), corresponding to that which is found in the compartment furthest from the blood vessel, as well as in red for high oxygen concentration (10.5%), corresponding to that which is found in the compartment closest to the blood vessel. Solid lines represent sensitive cell growth rates, and dotted lines represent resistant cell growth rates. Examples of plasma concentration functions over time are shown for erlotinib in (B) and evofosfamide in (D). Given a standard dosing schedule of 150 mg erlotinib administered daily, the red curve in (B) shows the concentration of erlotinib found in the compartment closest to the nearest blood vessel, and the blue curve in (B) shows the erlotinib concentration found in the compartment furthest from the nearest blood vessel. (D) shows an example of a periodic function for evofosfamide plasma concentration over time, given a dosing schedule of 575 mg/m^2^ administered weekly.

Our recent experimental results have demonstrated that the response of non-small cell lung cancer tumor cells to erlotinib is dependent on the tumor microenvironment and, in particular, oxygen concentration. In [2], this dependence was quantified by obtaining live/dead cell counts for isogenic HCC827 sensitive and resistant (harboring the T790M resistance mutation) cell lines *in vitro* at varying concentrations of oxygen and drug. We then calculated birth and death rates as functions of oxygen and erlotinib concentration using an exponential growth model. Please see S1 Text for a detailed description of these functions. For each compartment, by setting the oxygen concentration in these functions equal to the oxygen concentration in that compartment, we obtain birth and death rates of sensitive and resistant cells as functions of erlotinib concentration. For example, Fig 2A shows the net growth rates of sensitive and resistant cells as functions of erlotinib concentration at a low oxygen concentration (0.33%) as well as a high oxygen concentration (10.5%). These values correspond to the concentrations of oxygen in the compartment furthest from the blood vessel and closest to the blood vessel, respectively. We observe that overall drug response for both cell types is more pronounced in high oxygen compartments than in low oxygen compartments, which actually comprise the tumor bulk. Strikingly, the resistant cells display essentially complete resistance under low oxygen levels but only partial resistance under high oxygen levels, whereas the sensitive cells exhibit only a minor increase in drug tolerance under low-oxygen levels. During the course of treatment with erlotinib, we let the birth and death rates of the sensitive cell population in compartment *i* be denoted by $\lambda_{X,i}^{1}\left(t\right)$ and $\mu_{X,i}^{1}\left(t\right)$, and we let $\lambda_{Y,i}^{1}\left(t\right)$ and $\mu_{Y,i}^{1}\left(t\right)$ represent the birth and death rates of the resistant cell population in compartment *i*.

*Analysis of erlotinib pharmacokinetic data.* During treatment the concentration of erlotinib within each compartment fluctuates in time, according to the dosing schedule. Assume erlotinib is administered in doses of *D*_1_ mg every *T*_1_ hours. We first determine how the drug concentration varies at the blood vessel over the course of treatment using clinical pharmacokinetic data [21, 41, 42]. The plasma concentration after one dose of erlotinib as a function of time is defined as:

$$\rho_{1}\left(t\right)=\left(7D_{1}+365\right)e^{-0.0307t}.$$ (4)

The details of this function derivation and fit are outlined in S1 Text. Using this function for plasma concentration after one dose of erlotinib, we define a function to describe plasma concentration over time for the entire course of treatment with erlotinib. The concentration of erlotinib at the blood vessel at some time *t* ∈ [*nT*_1_, (*n* + 1)*T*_1_) for *n* = 0, 1, 2, … is given by

$$C_{1}\left(t\right)=\sum_{i=0}^{n}\rho_{1}\left(t-iT_{1}\right).$$ (5)

Erlotinib concentration decays spatially away from the vessel exponentially. Since experimental characterization of erlotinib drug penetration in solid tumors is unavailable currently, this decay rate is parameterized based on the decay rate of doxorubicin, which has a similar molecular weight to erlotinib [3]; in [2] we investigated a range of decay rates to account for differences in cellular uptake rate between these two molecules. Combining this decay rate with *C*_1_(*t*) fully specifies a new function *C*_1,*i*_(*t*) for the concentration of erlotinib in each compartment *i* at time *t*. For example, the concentration of erlotinib over time, given a standard dosing schedule of 150 mg erlotinib daily, is plotted for two different compartments (the compartments closest to and furthest from the nearest blood vessel) in Fig 2B.

By substituting *C*_1,*i*_(*t*) into these expressions, we obtain functions describing the birth and death rates of sensitive and resistant cells in compartment *i* at any time *t* during treatment:

$$\begin{array}{cc} \lambda_{X,i}^{1}\left(t\right) & =\lambda_{X,i}^{1}\left(C_{1,i}\left(t\right)\right),\\ \mu_{X,i}^{1}\left(t\right) & =\mu_{X,i}^{1}\left(C_{1,i}\left(t\right)\right),\\ \lambda_{Y,i}^{1}\left(t\right) & =\lambda_{Y,i}^{1}\left(C_{1,i}\left(t\right)\right),\\ \mu_{Y,i}^{1}\left(t\right) & =\mu_{Y,i}^{1}\left(C_{1,i}\left(t\right)\right). \end{array}$$ (6)

#### Effect of evofosfamide and oxygen on growth kinetics

Next we consider the effect of evofosfamide on the erlotinib-sensitive and erlotinib-resistant cell populations in each microenvironmental compartment. Note that the mechanisms of action differ greatly between erlotinib and evofosfamide. In particular, the mutation conferring resistance to erlotinib occurs in the EGFR kinase domain which is independent of the action of evofosfamide; thus the presence of this mutation is not thought to impact evofosfamide response. So we assume the same response of erlotinib-sensitive and erlotinib-resistant cells to evofosfamide. The net growth rate dependence on evofosfamide concentration in each microenvironmental compartment is calculated from cell viability experiments performed in [19]. These functions are plotted in Fig 2C for a low oxygen concentration (0.33%) and a high oxygen concentration (10.5%). Since evofosfamide releases a cytotoxic agent in hypoxic regions, we assume that its primary effect is to increase cellular death rate rather than to decrease cellular birth rates. Using this assumption, birth and death rates are derived from the net growth rates. For a detailed outline of the derivation of these birth and death rates, see S1 Text. In the following we denote the birth and death rates of sensitive cells in compartment *i* during treatment with evofosfamide by $\lambda_{X,i}^{2}\left(t\right)$ and $\mu_{X,i}^{2}\left(t\right)$, and we let $\lambda_{Y,i}^{2}\left(t\right)$ and $\mu_{Y,i}^{2}\left(t\right)$ represent the birth and death rates of resistant cells in compartment *i*.

*Analysis of evofosfamide pharmacokinetic data.* Next we consider how the concentration of evofosfamide changes during treatment. The plasma concentration after a dose of *D*_2_ mg/m^2^ of evofosfamide as a function of time is given by:

$$\rho_{2}\left(t\right)=\left\{\begin{array}{cc} 2c_{2}^{max}\left(D_{2}\right)t & t<\frac{1}{2}\\ c_{2}^{max}\left(D_{2}\right)e^{-k_{2}\left(D_{2}\right)\left(t-1/2\right)} & t\geq \frac{1}{2}. \end{array}\right.$$ (7)

Here, the functions describing maximum plasma concentration and decay rate in terms of dose are given by

$$\begin{array}{ll} c_{2}^{max}(D_{2}) & =(2.008\cdot 10^{-7})D_{2}^{3}-0.0003276D_{2}^{2}+0.1753D_{2}-12.54,\\ k_{2}(D_{2}) & =(1.302\cdot 10^{-7})D_{2}^{3}-0.0002043D_{2}^{2}+0.08873D_{2}-5.829. \end{array}$$

The interested reader should consult S1 Text for the details of this derivation, using pharmacokinetic data from a phase 1 clinical trial [43]. Since evofosfamide is very quickly eliminated from the blood stream (half-life of 0.81 hours [43]), for treatment schedules where doses are spaced at least 6 hours apart, the plasma concentration over the course of treatment *C*_2_(*t*) can be approximated as a periodic function. Then for *n* = 0, 1, 2, … and *t* ∈ [*nT*_2_, (*n* + 1)*T*_2_), where *T*_2_ is the number of hours between doses and *T*_2_ ≥ 6, the concentration of evofosfamide at the blood vessel at time *t* is given by

$$C_{2}\left(t\right)=\rho_{2}\left(t-nT_{2}\right).$$ (8)

Fig 2D shows the plasma concentration function corresponding to a dosing schedule of 575 mg/m^2^ evofosfamide given every week.

Next we consider how the birth and death rates of sensitive and resistant cells change across environmental compartments at various oxygen levels. Experimental studies have quantified the distribution of apoptotic and proliferative markers within tumor tissue after treatment with evofosfamide and showed relatively uniform expression levels with respect to distance from the nearest blood vessel [44]. This suggests that cell birth and death are also approximately uniform in these compartments until one reaches the more hypoxic regions of the tumor where apoptosis and DNA damage levels increase. Therefore, unlike with erlotinib, we assume the concentration of evofosfamide in every compartment of the tumor microenvironment at any given time is equal to the concentration *C*_2_(*t*) at the blood vessel at that time.

Finally, using *C*_2_(*t*) we obtain birth and death rates of sensitive and resistant cells in each compartment *i* at any time *t* during treatment:

$$\begin{array}{cc} \lambda_{X,i}^{2}\left(t\right) & =\lambda_{X,i}^{c},\\ \mu_{X,i}^{2}\left(t\right) & =\mu_{X,i}^{c}-\frac{1}{2}\ln\left[v_{i}\left(C_{2}\left(t\right)\right)\right],\\ \lambda_{Y,i}^{2}\left(t\right) & =\lambda_{X,i}^{c},\\ \mu_{Y,i}^{2}\left(t\right) & =\mu_{Y,i}^{c}-\frac{1}{2}\ln\left[v_{i}\left(C_{2}\left(t\right)\right)\right]. \end{array}$$ (9)

In the above equations, the superscript *c* denotes the control growth rates in the absence of drug. These are defined in the first section of S1 Text. *v*_*i*_ is the function describing cell viability in terms of evofosfamide concentration, as defined in S1 Text.

#### Growth kinetics during Combination therapy

Consider a combination dosing regimen consisting of some number of identical cycles, each of length *t*_1_ + *t*_2_ hours. During each cycle, assume the first *t*_1_ hours are dedicated to treatment with erlotinib, whereas the last *t*_2_ hours are used for evofosfamide treatment. For the reasons discussed above, given a sufficient amount of time between the last dose of evofosfamide in one cycle and the first dose of erlotinib in the next cycle, we may assume that during the erlotinib treatment phase there is no residual evofosfamide. Therefore the birth and death rates during the first *t*_1_ hours in every cycle are governed by erlotinib response kinetics. Since erlotinib has a much longer half-life than evofosfamide, during the last *t*_2_ hours of evofosfamide treatment it is possible that some erlotinib will remain in the blood stream by the beginning of this period. Therefore the cellular birth and death rates must reflect responses to both drugs. However, since erlotinib is primarily cytostatic while evofosfamide is primarily cytotoxic, we assume that during this period of time, cellular birth rates reflect the response to erlotinib while cellular death rates reflect the response to evofosfamide.

Since cycles are identical, functions describing birth and death rates over the course of treatment are periodic with period *t*_1_ + *t*_2_. Using the equations in Eqs (6) and (9), we define birth and death rates of the sensitive and resistant cell populations in compartment *i* at time *t* ∈ [0, *t*_1_ + *t*_2_) by

$$\begin{array}{cc} \lambda_{X,i}\left(t\right) & =\lambda_{X,i}^{1}\left(C_{1,i}\left(t\right)\right),\\ \mu_{X,i}\left(t\right) & =\left\{\begin{array}{cc} \mu_{X,i}^{1}\left(C_{1,i}\left(t\right)\right) & t\in [0,t_{1})\\ \mu_{X,i}^{c}-\frac{1}{2}\ln\left[v_{i}\left(C_{2}\left(t\right)\right)\right] & t\in [t_{1},t_{1}+t_{2}), \end{array}\right.\\ \lambda_{Y,i}\left(t\right) & =\lambda_{Y,i}^{1}\left(C_{1,i}\left(t\right)\right),\\ \mu_{Y,i}\left(t\right) & =\left\{\begin{array}{cc} \mu_{Y,i}^{1}\left(C_{1,i}\left(t\right)\right) & t\in [0,t_{1})\\ \mu_{Y,i}^{c}-\frac{1}{2}\ln\left[v_{i}\left(C_{2}\left(t\right)\right)\right] & t\in [t_{1},t_{1}+t_{2}). \end{array}\right. \end{array}$$ (10)

These functions are used in Eqs (1), (2) and (3) to predict tumor evolutionary dynamics over the course of treatment.

#### Model parameter notations

We have summarized the parameters used in our model in Table 1. These parameters are listed in order of appearance in the paper. The first column in this table provides a variable name (if given in the paper) for the parameter and the second column provides its biological meaning. The third column gives the value of the constant parameters in the model.

**Table 1:** Model parameter notations. The parameters used in our model are summarized in the table below, in order of appearance in the paper. The first column contains the variable name of the parameter, if applicable. The second column summarizes the biological meaning of this parameter. The third column gives the units as well as values for any parameters which are constant in the model. The subscript *i* represents quantities in compartment *i*.

Name	Biological Meaning	Value and Units
–	oxygen decay rate (spatial)	−0.0385
*λ*_*X*,*i*_(*t*)	sensitive cell birth rate	hours^-1^ (see Eq (10))
*μ*_*X*,*i*_(*t*)	sensitive cell death rate	hours^-1^ (see Eq (10))
*λ*_*Y*,*i*_(*t*)	resistant cell birth rate	hours^-1^ (see Eq (10))
*μ*_*Y*,*i*_(*t*)	resistant cell death rate	hours^-1^ (see Eq (10))
*u*	mutation rate	10^−7^
*M*	initial number of sensitive cells in tumor	1.6 ⋅ 10^6^
*M*_*i*_	initial number of sensitive cells	(from compartment weights)
$\lambda_{X,i}^{1}\left(t\right)$	sensitive cell birth rate due to erlotinib	hours^-1^ (see Eq (6))
$\mu_{X,i}^{1}\left(t\right)$	sensitive cell death rate due to erlotinib	hours^-1^ (see Eq (6))
$\lambda_{Y,i}^{1}\left(t\right)$	resistant cell birth rate due to erlotinib	hours^-1^ (see Eq (6))
$\mu_{Y,i}^{1}\left(t\right)$	resistant cell death rate due to erlotinib	hours^-1^ (see Eq (6))
*D*_1_	dose of erlotinib	mg
*T*_1_	time between erlotinib doses	hours
*ρ*_1_(*t*)	plasma concentration after one dose erlotinib	ng/mL (see Eq (4))
*C*_1_(*t*)	plasma concentration during erlotinib treatment	ng/mL (see Eq (5))
–	erlotinib decay rate (spatial)	−0.0173
*C*_1,*i*_(*t*)	erlotinib concentration in compartment *i*	*μ*M (calculated using *C*_1_(*t*) and *i*)
$\lambda_{X,i}^{2}\left(t\right)$	sensitive cell birth rate due to evofosfamide	hours^-1^ (see Eq (9))
$\mu_{X,i}^{2}\left(t\right)$	sensitive cell death rate due to evofosfamide	hours^-1^ (see Eq (9))
$\lambda_{Y,i}^{2}\left(t\right)$	resistant cell birth rate due to evofosfamide	hours^-1^ (see Eq (9))
$\mu_{Y,i}^{2}\left(t\right)$	resistant cell death rate due to evofosfamide	hours^-1^ (see Eq (9))
*D*_2_	dose of evofosfamide	mg/m^2^
*T*_2_	time between evofosfamide doses	hours
*ρ*_2_(*t*)	plasma concentration after one dose evofosfamide	*μ*g/mL (see Eq (7))
*C*_2_(*t*)	evofosfamide plasma concentration	*μ*g/mL (see Eq (8))
$\lambda_{X,i}^{c}$	sensitive cell control birth rate	hours^-1^ (see SI section 1)
$\mu_{X,i}^{c}$	sensitive cell control death rate	hours^-1^ (see SI section 1)
$\lambda_{Y,i}^{c}$	resistant cell control birth rate	hours^-1^ (see SI section 1)
$\mu_{Y,i}^{c}$	resistant cell control death rate	hours^-1^ (see SI section 1)
*v*_*i*_(*C*_2_)	cell viability due to evofosfamide	(see SI section 3)
*t*_1_	duration of erlotinib treatment	hours
*t*_2_	duration of evofosfamide treatment	hours

## Results

Using the stochastic model, we examined the evolutionary dynamics of a tumor undergoing therapy with a wide variety of dosing regimens using erlotinib and evofosfamide, both separately and in combination. We first developed a model for the toxicity constraints governing both single-agent and combination therapies, based on reported toxicities and side effects of the two drugs in Phase I/II trials. We then optimized combination treatment strategies in order to predict which types of dosing strategies could lead to the best treatment outcomes for patients diagnosed with non-small cell lung cancer.

### Toxicity Constraints

We defined the space of all tolerated single-agent and combination therapy dosing schedules using clinical trial data on drug tolerability. For each single-agent treatment we defined a toxicity constraint curve representing the relationship between frequency of drug administration and maximum tolerated dose. In addition, we analyzed the overlapping toxicities between the two drugs as well as each drug elimination rate to determine the necessary conditions for safely administering both drugs in succession. This combination therapy constraint, together with the toxicity constraint curves corresponding to each of the monotherapies, defines the space of all tolerated dosing schedules.

#### Monotherapy toxicity constraints

For each drug, we derive a toxicity constraint curve such that all points on and below the curve correspond to tolerated dosing schedules, whereas all points above the curve correspond to dosing schedules which lead to dose-limiting toxicities. These curves are constructed using data from clinical trials on the tolerability and toxicity of each drug. For each dosing schedule tested in clinical trials, we convert the schedule to an ordered pair describing the relationship between frequency of drug administration and dose as follows: the first coordinate is defined to be the number of times the drug is administered in a 3-week period, and the second coordinate is given by the corresponding dose. The location of each ordered pair relative to the toxicity constraint curve is determined based on whether or not that dosing schedule was tolerated in clinical trials. Each of these clinically tested dosing schedules is listed in Table 2, along with its corresponding ordered pair and location relative to the toxicity constraint curve. Note that in order to handle the discrepancy between clinical trial data sets for the 200 mg/day erlotinib dosing schedule, we err on the side of caution and assume this dosing schedule is not tolerated.

**Table 2:** Drug tolerability data from clinical trials. Details regarding erlotinib and evofosfamide dosing schedules tested in clinical trials and how this data informs the construction of the toxicity constraint curve for each drug. The dose administered is shown in the first column, and the corresponding dosing schedule is shown in the second column. The third column shows whether or not this particular dosing schedule was tolerated in the clinical trial. The fourth column shows the ordered pair this dosing schedule corresponds to, and the last column shows the location of this point relative to the toxicity constraint curve. We assume that any point corresponding to a maximum tolerated dosing schedule tested in a clinical trial can either lie on or below the toxicity constraint curve to account for the possibility that a higher dose (which was not tested in the trial) is tolerated.

**Erlotinib**				
Dose (mg)	Schedule	Tolerated?	(*n*,*D*_1_)	Location
25	daily for 3 days/week	yes [45]	(9, 25)	below curve
50	daily for 3 days/week	yes [45]	(9, 50)	below curve
50	daily	yes [45]	(21, 50)	below curve
100	daily for 3 days/week	yes [45]	(9, 100)	on/below curve
100	daily	yes [41, 45]	(21, 100)	below curve
100	twice daily	no [45]	(42, 100)	above curve
150	daily	yes [41, 45]	(21, 150)	on/below curve
200	daily	yes [41], no [45]	(21, 200)	above curve
250	daily	no [41]	(21, 250)	above curve
1200	weekly	yes [46]	(3, 1200)	below curve
1600	weekly	yes [46]	(3, 1600)	below curve
2000	weekly	yes [46]	(3, 2000)	on/below curve
**Evofosfamide**				
Dose (mg/m^2^)	Schedule	Tolerated?	(*n*,*D*_2_)	Location
7.5	weekly	yes [43]	(3, 7.5)	below curve
15	weekly	yes [43]	(3, 15)	below curve
30	weekly	yes [43]	(3, 30)	below curve
60	weekly	yes [43]	(3, 60)	below curve
120	weekly	yes [43]	(3, 120)	below curve
120	5 days every 3 weeks	yes [47]	(5, 120)	below curve
170	5 days every 3 weeks	yes [47]	(5, 170)	below curve
240	weekly	yes [43]	(3, 240)	below curve
240	5 days every 3 weeks	yes [47]	(5, 240)	below curve
330	5 days every 3 weeks	yes [47]	(5, 330)	below curve
460	5 days every 3 weeks	yes [47]	(5, 460)	on/below curve
480	weekly	yes [43]	(3, 480)	below curve
550	5 days every 3 weeks	no [47]	(5, 550)	above curve
575	weekly	yes [43]	(3, 575)	on/below curve
670	weekly	no [43]	(3, 670)	above curve
670	once every 3 weeks	yes [43]	(1, 670)	on/below curve
940	once every 3 weeks	no [43]	(1, 940)	above curve

Using the data from Table 2, we construct toxicity constraint curves for erlotinib and evofosfamide. For each drug, we define a function to describe the relationship between maximum tolerated dose and frequency of drug administration such that the criteria in the last two columns of Table 2 are satisfied. The erlotinib toxicity constraint curve is defined as follows, where *n* is the number of times erlotinib is administered in a 3-week period and *D*_1_ represents the maximum tolerated erlotinib dose (in mg) corresponding to that particular dosing schedule:

$$D_{1}\left(n\right)=\{\begin{array}{ll} 2000 & n\leq 3\\ 2000e^{-0.1439(n-3)} & n\geq 3. \end{array}$$

The toxicity constraint curve for evofosfamide is given by the following function, where *n* represents the number of times evofosfamide is administered in a 3-week period and *D*_2_ is the maximum tolerated dose of evofosfamide (in mg/m^2^) associated to the dosing schedule defined by the value of *n*:

$$D_{2}\left(n\right)=\{\begin{array}{ll} 670 & n\leq 1\\ 670e^{-0.076454\left(n-1\right)} & n\geq 1. \end{array}$$

**Fig 3:**
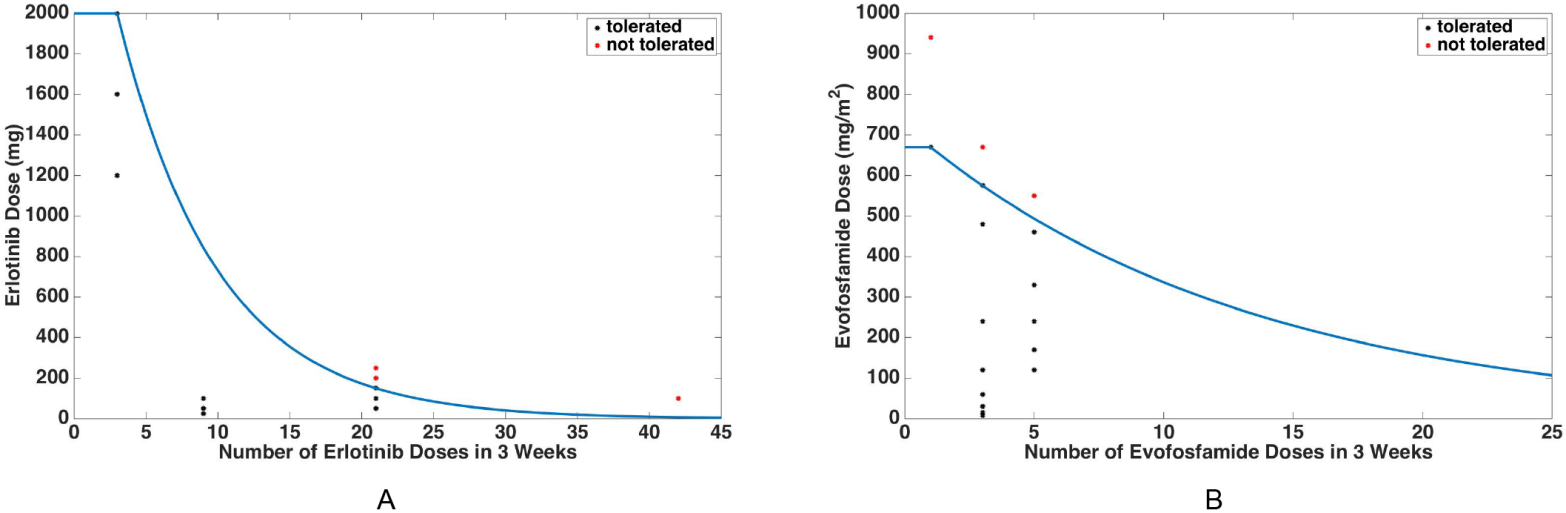
Toxicity constraint curves for erlotinib and evofosfamide. These curves depict the maximum tolerated doses of erlotinib (A) and evofosfamide (B) as functions of frequency of dose administration. The black points are the coordinates from Table 2 corresponding to tolerated dosing schedules, and the red points are the ordered pairs in Table 2 associated to dosing schedules that were not tolerated in clinical trials. All points contained in the areas on and below these two curves make up the space of tolerated monotherapy dosing schedules, and all points contained in the areas above these two curves make up the space of dosing schedules which lead to dose-limiting toxicities. The curves themselves represent the space of all monotherapy maximum tolerated dosing schedules.

These curves, along with the corresponding data points from Table 2, are shown in Fig 3.

#### Combination therapy toxicity constraints

To characterize the toxicity constraints relevant for considering alternating dosing combination strategies, we study the minimum length of time that should pass between the last dose of one drug and the first dose of the other drug. Since evofosfamide is eliminated from the blood stream in a matter of a few hours, we assume that as long as six or more hours have elapsed since the last dose of evofosfamide, a patient can safely take a dose of erlotinib without the worry of compounding side effects from the two drugs.

Next, we aim to determine the necessary waiting time between a last dose of erlotinib and a first dose of evofosfamide. Since the half-life of erlotinib is much longer than that of evofosfamide, it may not be reasonable to wait until erlotinib has been completely eliminated from the blood stream before administering evofosfamide, as waiting this long with so little drug in the blood stream could allow the cancer cells to rapidly proliferate. Analysis of clinical trial data for both drugs indicates that erotinib and evofosfamide can both cause skin and mucosal toxicities, but there are no other overlapping toxicities reported. When erlotinib is administered three days every week, skin toxicities are observable at a dose of 50 mg, but not a dose of 25 mg [45]. Similarly, when erlotinib is given daily, mucosal toxicities are observable at a dose of 150 mg, but not at 100 mg [45]. Hence we assume that a patient taking 25 mg erlotinib three days every week experiences neither skin nor mucosal toxicities. Given this dosing schedule, a maximum plasma concentration of 2.357 *μ*M is achieved, according to the previously defined erlotinib plasma concentration function. Thus we assume that once erlotinib plasma concentration has fallen to 2.357 *μ*M or lower, it is safe to administer evofosfamide since this concentration of erlotinib will not cause any side effects overlapping with those caused by evofosfamide. Although this is most likely a simplification of the true combination toxicity constraints, we feel it is a realistic assumption based on the single-agent toxicity data available.

### Comparison of combination strategies with standard monotherapy schedules

To investigate the potential of combination therapies, we first compare treatment outcomes resulting from several combination therapies with the monotherapy schedules currently in clinical use. The standard dosing schedule for erlotinib is 150 mg/day. Two evofosfamide dosing schedules have been tested in a clinical trial and designated as maximum tolerated dosing schedules: 670 mg/m^2^ given every 3 weeks and 575 mg/m^2^ given weekly. We consider combination schedules which are clinically feasible and satisfy the toxicity constraints described in the previous section. Table 3 provides an overview of all of these dosing schedules. Schedule A is the standard erlotinib dosing schedule, and schedules B and C are the two evofosfamide dosing schedules. The remaining schedules (D through J) represent all combination therapies considered in this analysis. Since the toxicity constraints for erlotinib and evofosfamide are formulated in terms of the number of doses administered in a 3-week period, we define these schedules based on 3-week cycles, and hence only show dosing protocols for the first 21 days since this pattern repeats every 3 weeks for each schedule. Entries in Table 3 represent doses of either erlotinib in mg (subscript 1) or evofosfamide in mg/m^2^ (subscript 2). For a fixed schedule (column) and day (row), a single entry represents the one dose scheduled for that day, and the lack of an entry indicates that no drugs are administered on that day. Two entries on a single day for a given schedule represent the scheduling of two doses on the same day.

**Table 3:** Dosing schedules considered in the comparison of single-agent and combination therapies. Each lettered column denotes a distinct dosing schedule containing repeating 3-week cycles defined by the dosing protocols in that column. The entries with subscripts of 1 are doses of erlotinib in mg and the entries with subscripts of 2 are doses of evofosfamide in mg/m^2^. For a fixed schedule (column) and day (row), a single entry represents the one dose of either erlotinib or evofosfamide scheduled for that day. A missing entry for a fixed schedule and day corresponds to a day with neither erlotinib nor evofosfamide. Two entries for a given schedule on a single day represent the scheduling of two doses, either one dose of each drug or two doses of the same drug.

Day	A	B	C	D	E	F	G	H	I	J
1	150_1_	670_2_	575_2_	150_1_	150_1_	7_1_ 7_1_	7_1_ 7_1_	150_1_	150_1_	150_1_ 145_2_
2	150_1_			150_1_	150_1_	7_1_ 7_1_	7_1_ 7_1_	150_1_	150_1_	150_1_ 145_2_
3	150_1_			150_1_	150_1_	7_1_ 7_1_	7_1_ 7_1_	150_1_	150_1_	150_1_ 145_2_
4	150_1_			150_1_	150_1_	7_1_ 7_1_	7_1_ 7_1_	150_1_	150_1_	150_1_ 145_2_
5	150_1_			150_1_	150_1_	7_1_ 7_1_	7_1_ 7_1_	150_1_	150_1_	150_1_ 145_2_
6	150_1_			150_1_		7_1_ 7_1_	7_1_ 7_1_	150_1_	150_1_	150_1_ 145_2_
7	150_1_			150_1_	575_2_	7_1_ 7_1_	575_2_	150_1_	575_2_	150_1_ 145_2_
8	150_1_		575_2_	150_1_	150_1_	7_1_ 7_1_	7_1_ 7_1_	150_1_	150_1_	150_1_ 145_2_
9	150_1_			150_1_	150_1_	7_1_ 7_1_	7_1_ 7_1_	150_1_	150_1_	150_1_ 145_2_
10	150_1_			150_1_	150_1_	7_1_ 7_1_	7_1_ 7_1_	150_1_	150_1_	150_1_ 145_2_
11	150_1_			150_1_	150_1_	7_1_ 7_1_	7_1_ 7_1_	150_1_	150_1_	150_1_ 145_2_
12	150_1_			150_1_	150_1_	7_1_ 7_1_	7_1_ 7_1_	150_1_	150_1_	150_1_ 145_2_
13	150_1_			150_1_		7_1_ 7_1_	7_1_ 7_1_	150_1_	150_1_	150_1_ 145_2_
14	150_1_			150_1_	575_2_	7_1_ 7_1_	575_2_	150_1_	575_2_	150_1_ 145_2_
15	150_1_		575_2_	150_1_	150_1_	7_1_ 7_1_	7_1_ 7_1_	150_1_	150_1_	150_1_ 145_2_
16	150_1_			150_1_	150_1_	7_1_ 7_1_	7_1_ 7_1_	150_1_	150_1_	150_1_ 145_2_
17	150_1_			150_1_	150_1_	7_1_ 7_1_	7_1_ 7_1_	150_1_	150_1_	150_1_ 145_2_
18	150_1_			150_1_	150_1_	7_1_ 7_1_	7_1_ 7_1_	150_1_	150_1_	150_1_ 145_2_
19	150_1_			150_1_	150_1_	7_1_ 7_1_	7_1_ 7_1_	150_1_	150_1_	150_1_ 145_2_
20	150_1_					7_1_	7_1_ 7_1_	150_1_	150_1_	150_1_ 145_2_
21	150_1_			670_2_	575_2_	670_2_	575_2_	670_2_	575_2_	150_1_ 145_2_

**Fig 4:**
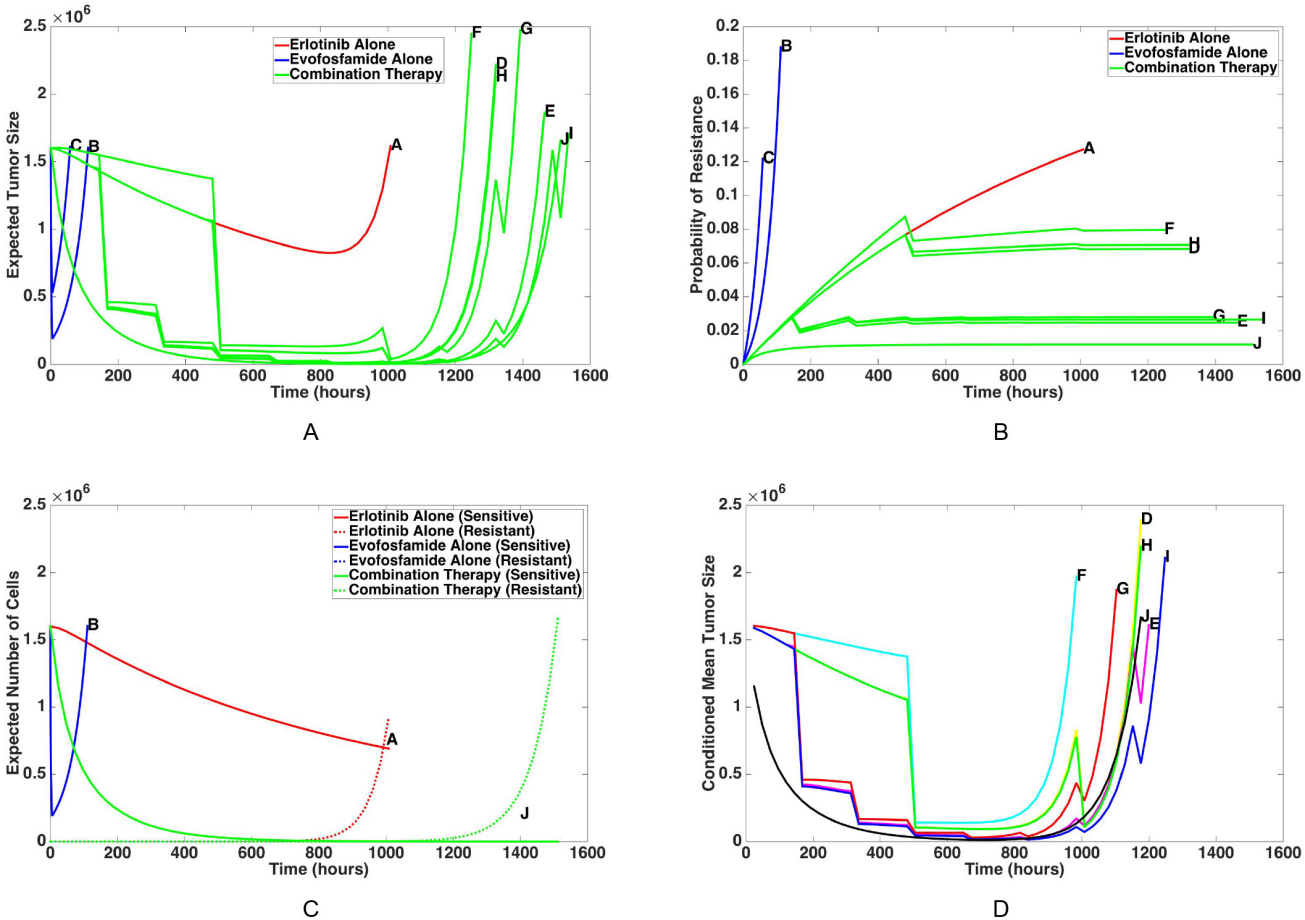
Tumor evolutionary dynamics over time, given a variety of single-agent and combination therapies. Mean tumor size (A) and probability of resistance (B) are calculated up to recurrence time for a tumor with an initial population of 1.6 ⋅ 10^6^ sensitive cells undergoing treatment with each of the ten dosing schedules defined in Table 3. Each labeled curve corresponds to the dosing schedule with the matching letter in Table 3. For the sake of comparison, results due to dosing schedules using erlotinib alone are shown in red, results due to dosing schedules using evofosfamide alone are shown in blue, and results due to combination therapies are shown in green. Mean tumor size for one of each of these three types of dosing schedules is broken down into the means of sensitive and resistant cells in (C). (D) shows the expected tumor size for combination strategies, conditioned upon the event of developing resistance.

For each of the ten dosing schedules in Table 3, the mean tumor size and probability of resistance over the course of treatment is predicted using the model. The results of these calculations up to recurrence time (the time at which the cancer cell population reaches its initial size once again) are plotted in Fig 4A and 4B, respectively. The red curves show the evolutionary dynamics of a tumor during treatment with erlotinib alone, the blue curves show the dynamics during monotherapy with evofosfamide, and the green curves show the evolutionary dynamics of the cancer cell population during combination therapy. The label on each curve indicates which dosing schedule from Table 3 corresponds to those results. The means of the sensitive and resistant cell populations are shown separately in Fig 4C for one of each type of dosing schedule: erlotinib alone, evofosfamide alone, and combination therapy. Fig 4D shows the mean tumor size for the combination schedules, averaged only over those that develop resistance.

We find that all the combination therapies considered produce treatment outcomes superior to the standard monotherapy schedules. Fig 4A demonstrates that the combination schedules result in lower average tumor sizes over the course of treatment than those resulting from either of the monotherapies. Even more significantly, Fig 4B shows that the probability of developing resistance decreases dramatically with the use of combination therapy. Under monotherapy with either drug, the probability of resistance eventually reaches one (in agreement with clinical results); this is due to the fact that sensitive cell division is not sufficiently inhibited by therapy to prevent the emergence of resistance before eradication of the tumor. However the model predicts that for a significant fraction of patients tumor eradication is possible under combination therapy.

The breakdown of sensitive and resistant cell populations under therapy is shown in Fig 4C. Erlotinib monotherapy yields a steady but slow decline of the sensitive cell population, due to the fact that the tumor oxygen distribution consists primarily of hypoxic regions and erlotinib does not penetrate well to these areas. On the other hand, treatment with evofosfamide alone targets hypoxic regions which comprise the majority of the cell population, leading to an initial steep decline of the sensitive cell population. However, due to the toxicity constraint during the subsequent break in treatment the mean of the sensitive cells quickly surpasses the initial population size and drives the production of erlotinib-resistant mutants. During combination therapy, however, the cancer cell population demonstrates an initial steep decline due to evofosfamide, followed by a long-term controlled phase due to the combination of evofosfamide and erlotinib. This tight control over the sensitive cell population during combination therapy is possible due to the fact that cancer cells close to blood vessels are receiving lethal concentrations of erlotinib while cancer cells in hypoxic regions are targeted by evofosfamide. Fig 4D demonstrates that for the patients who develop resistance, the average length of time until tumor recurrence is longer for all of the combination therapy dosing schedules than it is for either of the standard monotherapies. For example, it takes patients who develop resistance 40.54% longer to rebound on Schedule I compared with standard erlotinib therapy. However, the length of time until recurrence as well as the overall probability of resistance varies between specific combination schedules; this serves as motivation for identifying the optimal timing and dosage sequence for combination schedules in the following section. Finally, we note that the combination strategies were also compared to optimized monotherapies (subject to the toxicity constraints developed in the previous section), and we found that no tolerated monotherapy schedule could outperform combinations in delaying or preventing resistance.

### Optimized combination strategies

We next utilize the mathematical model to optimize over the space of tolerated combination treatment strategies (constrained by toxicity constraints derived in the previous section) to minimize the probability of developing resistance or maximally delay recurrence.

We consider three distinct classes of combination therapies. Class 1 investigates schedules created by systematically combining standard erlotinib monotherapy with a variety of evofosfamide dosing schedules. However, the amount of time between administration of different drugs can play an important role in the degree to which therapy affects the cancer cell population. Thus the rationale for defining the other two classes are to investigate schedules decreasing the amount of time after erlotinib but before evofosfamide dosing (Class 2), and after evofosfamide but before erlotinib dosing (Class 3).

For each class, we start with a base erlotinib dosing schedule complying with the monotherapy toxicity constraint curve in Fig 3A. Modifications to this schedule are then made to incorporate *n* doses of evofosfamide, where *n* varies from 0 (corresponding to erlotinib monotherapy) to a maximal value *N* (corresponding to evofosfamide monotherapy), in a three-week period. The dose of evofosfamide is determined by the toxicity constraint curve in Fig 3B. Whenever necessary, the minimum number of erlotinib doses are removed to comply with the combination toxicity constraint described in the previous section.

To define each combination schedule, we begin by defining a single cycle of length *L* = 504/*n* hours, consisting of the base erlotinib dosing schedule and a single dose of evofosfamide. This is done using a 4-step process: (i) calculate the evofosfamide dose, (ii) place the evofosfamide dose at either *t* = *L* − 24 or *t* = *L* − 6 depending on the class, (iii) fill the remaining time in the cycle with the base erlotinib dosing schedule, and (iv) remove any necessary erlotinib doses to comply with the combination toxicity constraint. For a detailed description of how to define a single cycle of treatment for all combination dosing schedules, see S1 Text. These cycles are then repeated some finite number of times to form complete dosing schedules. In Class 1, we use the standard erlotinib monotherapy schedule of 150 mg/day, and the evofosfamide dose in each cycle is given 24 hours before the start of the next cycle. In Class 2, we use a low-dose erlotinib schedule of 7 mg twice daily, which allows for a shorter waiting period after erlotinib doses and before evofosfamide doses. Class 3 uses the same standard erlotinib monotherapy as in Class 1; however, the evofosfamide dose in each cycle is given 6 hours before the start of the next cycle instead of 24, which decreases the amount of time after evofosfamide doses and before erlotinib doses.

**Fig 5:**
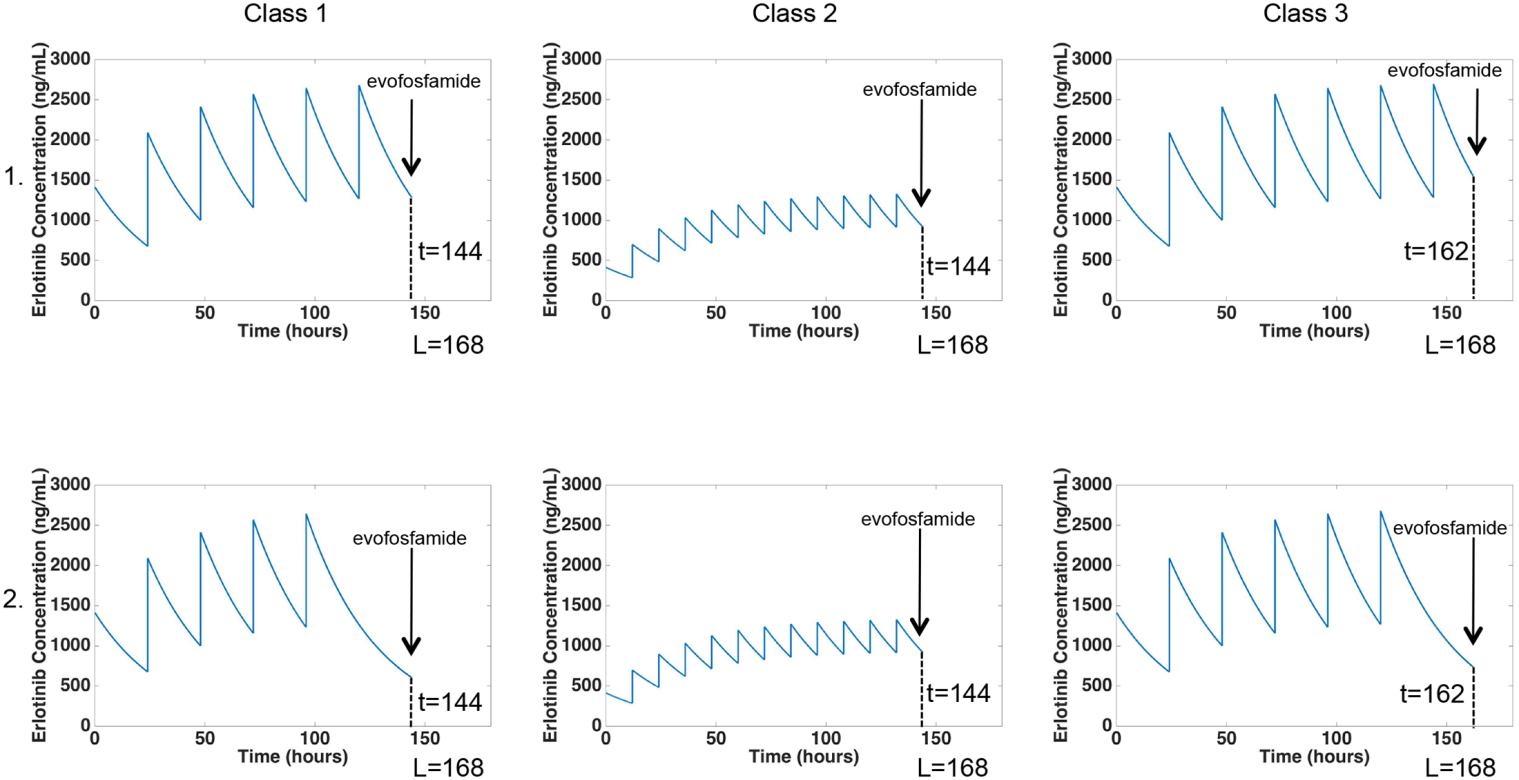
Example depicting dose schedule definition for one cycle of treatment with *n* = 3 for all optimization classes. This schematic shows the process by which one cycle of treatment is defined for each optimization class with *n* = 3. A cycle in Class 1 or 3 contains a standard erlotinib dosing schedule of 150 mg/day, whereas a cycle in Class 2 contains a low-dose erlotinib schedule of 7 mg twice daily. When *n* = 3, each cycle has length *L* = 168 (one week). For Classes 1 and 2, the evofosfamide dose in each cycle is given 24 hours before the end of the week, and for Class 3 the evofosfamide dose in each cycle is given 6 hours before the end of the week. This is all depicted in step 1. Step 2 shows the removal of erlotinib doses required to satisfy the combination toxicity constraint. Each of these cycles is then repeated to form the entire dosing schedule.

Fig 5 shows an example depicting dose schedule definition for one cycle of treatment for all three optimization classes when *n* = 3. For the reasons stated in the above paragraph, a cycle in Class 1 or 3 contains a standard erlotinib dosing schedule, whereas a cycle in Class 2 contains a low-dose erlotinib schedule. When *n* = 3, each cycle is one week. So for Classes 1 and 2, the evofosfamide dose in each cycle is given 24 hours before the end of the week, and for Class 3 the evofosfamide dose in each cycle is given 6 hours before the end of the week. This information is all depicted in step 1 (the top row) of Fig 5. In step 2 (the bottom row), the necessary number of erlotinib doses leading up to the evofosfamide infusion is removed in order to satisfy the combination toxicity constraint. In Classes 1 and 3, the last dose of erlotinib shown in step 1 is removed in step 2 to allow the erlotinib concentration to fall sufficiently low before the evofosfamide infusion. Note that in Class 2 the removal of erlotinib is not necessary due to the already low erlotinib concentration.

**Fig 6:**
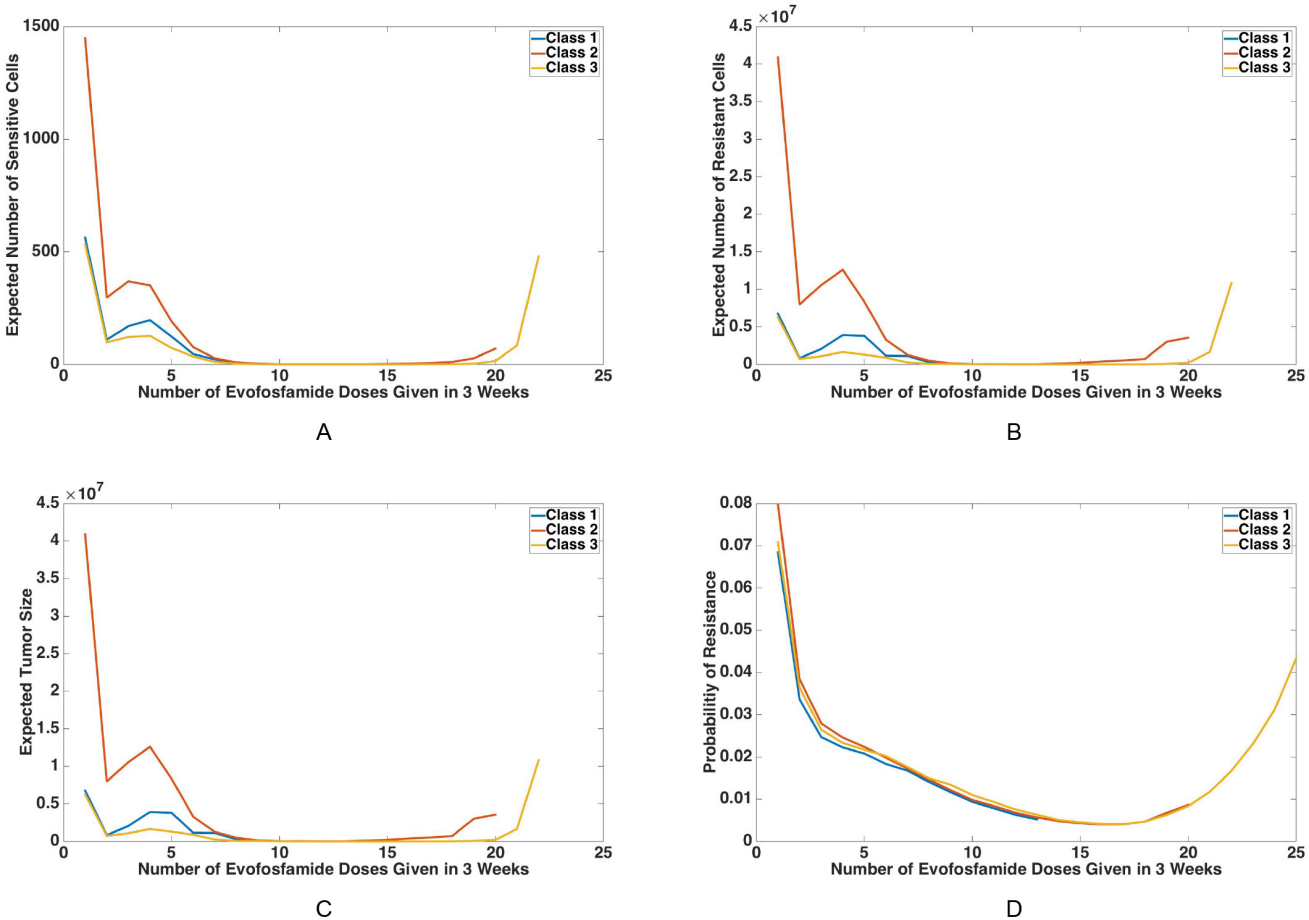
Probability of resistance and means of sensitive and resistant cells at the end of treatment with combination therapy. For every dosing schedule in each optimization class, means of the sensitive cells (A), resistant cells (B), and total tumor size (C), as well as probability of resistance (D), are calculated according to the model at the end of nine weeks of treatment for a tumor initially consisting of 1.6 ⋅ 10^6^ sensitive cells. The results shown here only include dosing schedules from Class 1 (blue), Class 2 (red), and Class 3 (yellow) which use a combination of both erlotinib and evofosfamide. Every integer on the *x*-axis represents a combination dosing schedule defined by the number of evofosfamide doses administered in three weeks.

*Combination schedules outperform monotherapy endpoints.* We calculated the means of the sensitive and resistant cells as well as the probability of resistance, after nine weeks of treatment for every dosing schedule in each optimization class. Table 4 demonstrates that the tumor population size under *n* = 0 or *N* (i.e. the monotherapies) after 9 weeks is *O*(10^10^) for any optimization class or either drug type, whereas Fig 6 demonstrates that each of the combination therapies considered yields a total population size of *O*(10^7^) or less. In fact, when comparing Table 4 to Fig 6 we observe that all of the combination therapies we have investigated yield a considerable benefit (in terms of tumor population sizes as well as probability of resistance) over any of the optimal monotherapies in each dosing class. Thus we do not include the endpoints corresponding to monotherapies in Fig 6 due to the large disparity in the sizes of these results. Furthermore, at the end of treatment with evofosfamide, the tumor primarily consists of sensitive cells, which agrees with our previous observation that evofosfamide is unable to control the sensitive cell population without erlotinib. Note that some of the means are unrealistically high for an *in vivo* setting (> 10^12^), due to the fact that the model is parameterized using *in vitro* growth rates.

**Table 4:** Probability of resistance and means of sensitive and resistant cells at the end of treatment with monotherapy. Means of the sensitive cells (E[X]), resistant cells (E[Y]), and total tumor size (E[X+Y]), as well as the probability of resistance (P[Y>0]), are calculated for a tumor with an initial population of 1.6 ⋅ 10^6^ sensitive cells at the end of nine weeks of treatment. The dosing schedules depicted here include both types of monotherapies (erlotinib alone and evofosfamide alone) from all three optimization classes.

**Class 1**				
	E[X]	E[Y]	E[X+Y]	P[Y>0]
Erlotinib	5 ⋅ 10^5^	10^10^	10^10^	0.16
Evofosfamide	9 ⋅ 10^15^	7 ⋅ 10^9^	9 ⋅ 10^15^	1
**Class 2**				
	E[X]	E[Y]	E[X+Y]	P[Y>0]
Erlotinib	10^6^	4 ⋅ 10^10^	4 ⋅ 10^10^	0.22
Evofosfamide	3 ⋅ 10^17^	3 ⋅ 10^11^	3 ⋅ 10^17^	1
**Class 3**				
	E[X]	E[Y]	E[X+Y]	P[Y>0]
Erlotinib	5 ⋅ 10^5^	10^10^	10^10^	0.16
Evofosfamide	6 ⋅ 10^18^	10^13^	6 ⋅ 10^18^	1

Means of the sensitive and resistant cell populations for *n* = 1 … *N* − 1 (true combination therapies) are plotted in Fig 6A and 6B, respectively. The sums of these means, or the mean tumor sizes, are plotted in Fig 6C. Note the presence of two local minima in these three figures. This phenomenon is explained in S1 Text. The probability of resistance for each dosing schedule is plotted in Fig 6D. All four panels include results for Class 1 in blue, results for Class 2 in red, and results for Class 3 in yellow. Note that in these figures, every integer on the *x*-axis corresponds to a combination dosing schedule given by the number of evofosfamide doses in a 3-week period, as defined previously.

*Minimize treatment break after evofosfamide dosing.* Next consider the results due to combination therapy shown in Fig 6. We note that all three classes lead to similar results, which suggests that our findings regarding the characteristics of optimal combination dosing strategies are quite robust. Upon closer examination, Fig 6 demonstrates that the tumor population sizes under the Class 3 schedules are generally less than half the population sizes under Class 2, and also less than those under Class 1. This suggests that designing schedules that minimize the amount of time after a dose of evofosfamide and before a dose of erlotinib may lead to better control of the tumor population. This finding is in agreement with our previous observations that the tumor population response to evofosfamide is strong but short-lived; hence quickly intervening in the subsequent population growth phase is important.

As we move along the spectrum of combination densities (horizontal axis) from monotherapy with erlotinib to monotherapy with evofosfamide, there is a clear region in the interior (approximately *n* = 9–17 evofosfamide doses) where the tumor size and sensitive and resistant population size are minimized. This region also contains the region minimizing the probability of developing resistance. To investigate this further, Table 5 shows the specfic *n* which optimizes the given characteristic (means of sensitive, resistant, and total cancer cells as well as probability of resistance are all minimized). In addition, the bottom row of Table 5 indicates the best overall dosing schedule among all three classes which minimizes the particular value that column represents.

**Table 5:** Optimal dosing schedules for each class. For each class, this table shows the values of *n* for which the means of sensitive, resistant, and total cancer cells, as well as probability of resistance, are each minimized. For each column, the bottom row indicates which of the three classes produces the best overall result for that characteristic of the cancer cell population at the end of treatment.

	E[X]	E[Y]	E[X+Y]	P[Y>0]
Class 1	*n* = 13	*n* = 13	*n* = 13	*n* = 13
Class 2	*n* = 12	*n* = 12	*n* = 12	*n* = 16
Class 3	*n* = 14	*n* = 14	*n* = 14	*n* = 17
Best Overall	Class 3, *n* = 14	Class 3, *n* = 14	Class 3, *n* = 14	Class 2, *n* = 16

*Sequential alternating sequences are optimal.* From Table 5 we observe that all optimal dosing schedules correspond to values of *n* between 12 and 17. This implies that combination therapies incorporating more frequent, smaller doses of evofosfamide result in better treatment outcomes. Even more interestingly, all values of *n* in Table 5 correspond to the same type of dosing schedule. Besides *n* = 12 in Class 2, every other optimal *n* corresponds to a dosing schedule which alternates between a single dose of erlotinib and a single dose of evofosfamide. We call these alternating dosing schedules. The dosing schedule corresponding to *n* = 12 in Class 2 consists of two low doses of erlotinib for every dose of evofosfamide, which is still quite similar to the alternating dosing schedules. Thus, even though the optimization ranged over a full spectrum of treatment schedules incorporating variable dose densities for each drug, the optimal therapies were those that utilized close to an equal number of doses of evofosfamide and erlotinib in a sequential alternating fashion.

## Discussion

In this work we considered an approach to investigate the use of hypoxia-activated prodrugs (HAPs) to enhance the effectiveness of targeted therapies and in particular, prevent the (usually inevitable) emergence of drug resistance. To this end we developed a model reflecting the heterogeneity of oxygen and drug concentrations throughout a tumor to describe the evolutionary dynamics of resistance emerging under combination HAP-targeted therapy strategies. The model was parametrized using experimental and clinical pharmacokinetic data to investigate potential combinations of the HAP evofosfamide with the targeted tyrosine kinase inhibitor erlotinib against EGFR-activated non small cell lung cancer. Our model predictions are useful in comparing the outcomes of a spectrum of dosing schedules in the *in vitro* setting, and provide a means to use available experimental data to help inform and guide future clinical studies *in vivo*.

We investigated combinations in which doses were not given simultaneously (to avoid toxicities) and our model predicted that the complementary action of evofosfamide and erlotinib results in a combined ability to control the tumor’s evolution and growth. In particular:

1. Combination therapies outperform standard clinical monotherapies. This is most significantly realized in reduction of the probability of developing resistance. The time to progression, for those who develop resistance, is 40.54% longer using an optimal combination therapy rather than standard monotherapy with erlotinib.
2. Sequentially alternating single doses of each drug leads to minimal tumor burden and maximal reduction in probability of developing resistance. Deviating significantly from an equal number of evofosfamide and erlotinib doses leads to an increase in both average tumor burden and the probability of developing resistance.
3. Strategies minimizing the length of time after an evofosfamide dose and before erlotinib confer further benefits in reduction of tumor burden. The tumor population response to evofosfamide is strong but short-lived; hence quickly intervening in the subsequent population growth phase is important.

These alternating dosing schedules (and other similar dosing schedules) are likely the most effective because the constant switching between erlotinib and evofosfamide allows the strengths of these drugs to complement one another. Too much time spent taking erlotinib without evofosfamide allows the sensitive cell population to remain quite substantial for a long period of time (due to the lack of targeting the hypoxic regions), which, in turn, leads to a high probability of a resistance mutation arising. On the other hand, too much time spent on evofosfamide without erlotinib allows the sensitive cell population to expand drastically since evofosfamide is unable to control its long-term growth. Alternating between these two drugs allows each one to provide the necessary control over the cancer cell population the other one is lacking. In addition, it is important to consider the subpopulation of cancer cells each drug acts on. Erlotinib acts primarily on portions of the tumor microenvironment close to blood vessels, whereas evofosfamide acts primarily on hypoxic regions that are further from the blood stream. Because of this, alternating frequently between the two drugs allows the entire population of cancer cells in the tumor microenvironment to be constantly controlled by the drugs. This same phenomenon has recently been observed with a different combination therapy utilizing evofosfamide in neuroblastoma and rhabdomyosarcoma preclinical models [48].

These results demonstrate that incorporating HAPs in combination with targeted therapies may be an effective tool in preventing resistance, and suggest an alternative use for HAPs. Current clinical trials have combined HAPs not with targeted therapies but with chemotherapies to control tumor growth. In addition, these trials used dosing strategies involving simultaneous drug administration rather than sequential administration, as is used in our model. It is difficult to draw conclusions about the outcome of these clinical trials using our model since this would require growth rate parametrizations and pharmacokinetics for the chemotherapies utilized in the combination treatment (gemcitabine and doxorubicin) in different cell types (pancreatic cancers and soft tissue sarcomas). However, we did observe that the reduction in probability of developing resistance is dependent on the exact timing and sequence of the combination therapy. This highlights the importance of using mathematical modeling to predict treatment outcomes and inform decisions regarding schedules to be tested in clinical trials.

In addition to its promising clinical implications, this work provides insight into the biological factors which can cause a treatment strategy to either succeed or fail. Specifically, analysis and comparison of the tumor evolutionary dynamics during single-agent and combination therapy suggests that erlotinib and evofosfamide may be effective together because they target separate subpopulations within the tumor microenvironment and on much different scales of time with differing degrees of strength. This theory can be generalized to predict which types of drugs have the potential to be strong partners in combination therapy; specifically, this methodology can be applied to determine the biological and pharmacokinetic parameters that may lead to treatment success or failure with monotherapy or combination therapy. These findings highlight the importance of designing combination therapies with drugs whose strengths complement each other in order to maximize the therapeutic benefits. Another important implication of this work, and something to consider when designing combination dosing regimens using two or more drugs, is the role that variability in timing between the dosing of different drugs plays in treatment outcomes.

This work gave rise to multiple promising improvements that could be made in the treatment of non-small cell lung cancer. However, the dosing strategies proposed here need to be tested *in vivo* to verify these model predictions. In addition, this work provided a novel framework for defining drug toxicity constraints, which is sufficiently general to be extended to any drug or combination of drugs. One planned extension of this work is to further study the implications of cellular migration within the microenvironment on the evolutionary dynamics of the tumor. An initial investigation into this impact is shown in Section 7 of S1 Text. For this study, experimental work investigating the details of the migration patterns and quantification of migration rates in this system are necessary. Other extensions of this work include considering the possibility of pre-existing resistance as well as modeling the bystander effect, which refers to the idea that evofosfamide, once activated in a hypoxic region of the tumor, diffuses outward and affects cancer cells in normoxic regions as well [16, 19]. In addition, it would be useful to explore the effect of HAPs other than evofosfamide on the probability of developing resistance in order to determine whether the results presented here are specific to evofosfamide or rather are a general phenomenon of HAPs used in combination with tyrosine kinase inhibitors. Since evofosfamide is hypoxia-activated and birth and death rates due to erlotinib are microenvironment-dependent, there is good reason to suspect that alterations to the tumor microenvironment would have a large impact on treatment outcomes with both single-agent and combination therapy.

## Supporting Information

S1 Text Additional details. Includes additional details pertaining to some derivations, definitions, and results in the manuscript. Specifically, this includes details regarding the erlotinib and evofosfamide plasma concentration function fits. Also included is the derivation of birth and death rates in response to evofosfamide. The definition of a treatment cycle for the combination dosing strategies is given in full detail here. Additional analysis of the optimization results is also provided. A section evaluating the impact of incorporating migration between compartments is included.
(PDF)
