# Supplementary material for: Leveraging Hypoxia-Activated Prodrugs to Prevent Drug Resistance in Solid Tumors
Source: PLoS Comput Biol. 2016 Aug 25;12(8):e1005077. doi: 10.1371/journal.pcbi.1005077 (PMC4999195; doi:10.1371/journal.pcbi.1005077)
Supplement: S1 Text — Includes additional details pertaining to some derivations, definitions, and results in the manuscript. Specifically, this includes details regarding the erlotinib and evofosfamide plasma concentration function fits. Also included is the derivation of birth and death rates in response to evofosfamide. The definition of a treatment cycle for the combination dosing strategies is given in full detail here. Additional analysis of the optimization results is also provided. A section evaluating the impact of incorporating migration between compartments is included. (PDF) [file pcbi.1005077.s001.pdf]

SUPPORTING INFORMATION: LEVERAGING HYPOXIA-ACTIVATED PRODRUGS TO PREVENT DRUG RESISTANCE IN SOLID TUMORS

DANIKA LINDSAY, COLLEEN GARVEY, SHANNON MUMENTHALER, AND JASMINE FOO

1. Birth and death rates as functions of oxygen and erlotinib concentration

Here we provide functions approximating experimental data, first reported in [1], for the birth and death rates of erlotinib-sensitive and erlotinib-resistant cells in terms of oxygen concentration x and erlotinib concentration c_1. Given an erlotinib concentration of c_1 μM, let the index $I(c_1)$ be given by $I(c_1) = \lfloor 10c_1 + \frac{1}{2} \rfloor + 1$. Then the birth and death rate functions for sensitive and resistant cells are defined as follows:

$$\begin{aligned}
\lambda_X^1(x, c_1) &= SB_{I(c_1),1} + x \cdot SB_{I(c_1),2} + 1.217 \cdot SB_{I(c_1),3},\\
\mu_X^1(x, c_1) &= SD_{I(c_1),1} + x \cdot SD_{I(c_1),2} + 1.217 \cdot SD_{I(c_1),3},\\
\lambda_Y^1(x, c_1) &= RB_{I(c_1),1} + x \cdot RB_{I(c_1),2} + 1.217 \cdot RB_{I(c_1),3},\\
\mu_Y^1(x, c_1) &= RD_{I(c_1,1} + x \cdot RD_{I(c_1),2} + 1.217 \cdot RD_{I(c_1),3},
\end{aligned}$$

where SB, SD, RB, and RD are given by the matrices in the last section of this document. The "control" birth and death rates in the absence of drug in compartment i with oxygen concentration x_i are then given by:

$$\begin{aligned}
\lambda_{X,i}^c &= \lambda_X^1(x_i, 0),\\
\mu_{X,i}^c &= \mu_X^1(x_i, 0),\\
\lambda_{Y,i}^c &= \lambda_Y^1(x_i, 0),\\
\mu_{Y,i}^c &= \mu_Y^1(x_i, 0).
\end{aligned}$$

2. Derivation of erlotinib plasma concentration function

We define a function $\rho_1(t)$ describing plasma concentration over time after one dose of D_1 mg erlotinib using multiple sets of pharmacokinetic data. Assume the concentration of erlotinib decays exponentially as a function of time and let $\rho_1(t) = c_1^{max} e^{-k_1 t}$ describe this exponential decay, where c_1^{max} is the maximum plasma concentration achieved after one dose of erlotinib and k_1 is the rate of decay. Using available pharmacokinetic data, we acquire pairs of best-fit parameters for this function at a variety of doses between 100 and 300 mg. The values obtained for k_1 are averaged to produce $k_1 = 0.0307$, and a linear function $c_1^{max}(D_1) = 7D_1 + 365$ is defined to reflect the relationship between dose and maximum plasma

concentration. Hence we define erlotinib plasma concentration, given an initial dose D_1, as a function of time:

$$\rho_1(t) = (7D_1 + 365)e^{-0.0307t}.$$

3. Derivation of birth and death rates due to evofosfamide

Here we define functions for the birth and death rates of sensitive and resistant cells in terms of evofosfamide concentration. We start by defining the percent of viable cells $V_i(C_2)$ in compartment i as a function of evofosfamide concentration. Suppose that for a fixed concentration of oxygen, cell viability behaves as a logistic function with respect to drug concentration. Let $V(C_2) = 100 - \frac{A}{1+e^{-\alpha/\beta}} + \frac{A}{1+e^{(C_2-\alpha)/\beta}}$, with A, α, and β unknown parameters, to reflect the fact that cells are 100% viable in the absence of drug ($V(0) = 100$). We fit this logistic function to multiple experimentally-derived data sets for cell viability, each at a different fixed oxygen concentration between 0% and 21%, to obtain values for A, α, and β. The estimates of A are averaged to yield $A = 88.5983$, and the relationship between α and β for compartment i and x_i, the oxygen concentration in compartment i, is described as follows:

$$\alpha_i = \begin{cases} (1.937 \cdot 10^{-7})x_i^3 - (2.932 \cdot 10^{-6})x_i^2 + (1.252 \cdot 10^{-5})x_i + 3.97 \cdot 10^{-7} & x_i < 10\% \\ (2.613 \cdot 10^{-6})x_i - 4.727 \cdot 10^{-8} & x_i \geq 10\%, \end{cases}$$

$$\beta_i = \begin{cases} (6.389 \cdot 10^{-8})x_i^3 - (9.284 \cdot 10^{-7})x_i^2 + (3.87 \cdot 10^{-6})x_i + 3.792 \cdot 10^{-8} & x_i < 10\% \\ (6.366 \cdot 10^{-7})x_i - 3.421 \cdot 10^{-6} & x_i \geq 10\%. \end{cases}$$

Therefore, for compartment i, the percent of viable cells at an evofosfamide concentration of C_2 is expressed as

$$V_i(C_2) = 100 - \frac{A}{1 + e^{-\alpha_i/\beta_i}} + \frac{A}{1 + e^{(C_2-\alpha_i)/\beta_i}},$$

where A, α_i, and β_i are all defined as above. Fig. 1 shows a plot of this function together with the corresponding data points at the seven fixed oxygen concentrations examined in the cell viability experiments.

We now use experimental cell viability data together with the cell viability function to derive expressions $N_{X,i}(C_2)$ and $N_{Y,i}(C_2)$ for net growth rates of sensitive and resistant cells in compartment i as functions of evofosfamide concentration C_2. Let the fraction of viable cells in compartment i as a function of evofosfamide concentration be denoted by $v_i(C_2) = \frac{1}{100}V_i(C_2)$. Since we assume evofosfamide has the same effect on the sensitive and resistant cells, we need only define one function for net growth rate in compartment i in terms of evofosfamide concentration which we can then apply to both the sensitive and resistant cell populations. Let $N(x, d)$ represent the net growth rate of cells in an environment with oxygen concentration x (written as a percentage) and evofosfamide concentration d. We define this function by writing out explicit expressions for the number of cells in the treated and control groups at certain points in time according to the details of the cell viability experiments and comparing this with the cell viability function.

Let group A represent the control group in the cell viability experiments and let group B denote the group of cells treated with evofosfamide concentration C_2 at an

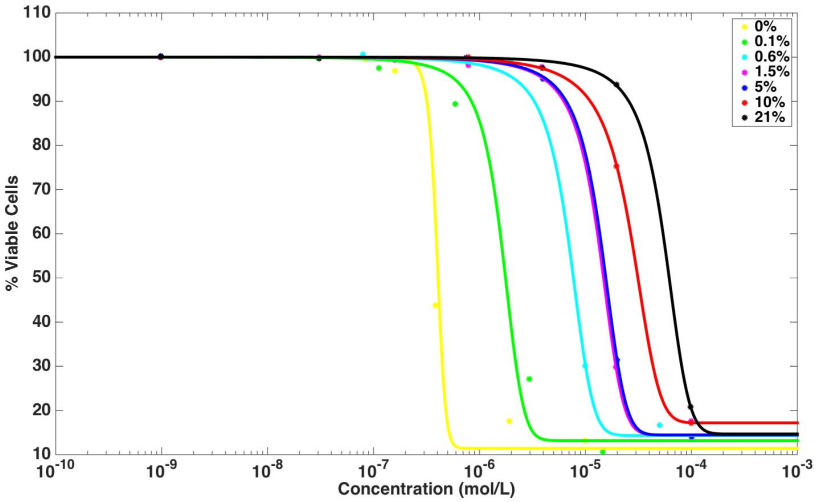

FIGURE 1. **Cell viability fit.** Percent of viable cells as a function of evofosfamide concentration. The points represent data from the cell viability experiments, and the curves show the fitted cell viability function at the fixed oxygen concentrations corresponding to the data.

oxygen concentration of x_i, corresponding to the oxygen concentration expected to coincide with compartment i of the tumor microenvironment. Use $n_A(t)$ and $n_B(t)$ to represent the number of cells in each of these groups at time t. Then for all t,

$$v_i(C_2) = \frac{n_B(t)}{n_A(t)}. \tag{1}$$

We use the detailed process followed in the cell viability experiments to write explicit expressions for the number of cells in groups A and B at certain points in time. At time $t = 0$, there are $n_A(0) = n_B(0)$ cells in each group. According to the cell viability data, exponentially growing cells were seeded 24 hours before the addition of test compounds. We express the number of cells present in each group at $t = 24$ by

$$n_A(24) = n_A(0)e^{24N(21,0)},$$
$$n_B(24) = n_B(0)e^{24N(21,0)}.$$

After the addition of evofosfamide, the plates were incubated for 2 hours under the altered oxygen concentration. The number of cells in each group at $t = 26$ is then given by

$$n_A(26) = n_A(24)e^{2N(x_i,0)},$$
$$n_B(26) = n_B(24)e^{2N(x_i,C_2)}.$$

Lastly, cells were cultured for 72 hours after washing under normoxic conditions. We can express the number of cells in each group at time $t = 98$ as

$$n_A(98) = n_A(26)e^{72N(21,0)},$$
$$n_B(98) = n_B(26)e^{72N(21,0)}.$$

By repeated substitution using the above equations, $n_A(98)$ and $n_B(98)$ can be written as follows:

$$\begin{aligned} n_A(98) &= n_A(0)e^{24N(21,0)}e^{2N(x_i,0)}e^{72N(21,0)}, \\ n_B(98) &= n_B(0)e^{24N(21,0)}e^{2N(x_i,C_2)}e^{72N(21,0)}. \end{aligned} \tag{2}$$

We proceed by relating the above equations to cell viability. Setting $t = 98$ in Eq. (1) yields

$$v_i(C_2) = \frac{n_B(98)}{n_A(98)}. \tag{3}$$

By substituting the equations in (2) into Eq. (3) and simplifying, keeping in mind that $n_A(0) = n_B(0)$, we obtain

$$v_i(C_2) = e^{2[N(x_i,C_2)-N(x_i,0)]}.$$

Taking the natural log of both sides, dividing by 2, and solving for $N(x_i, C_2)$ yields

$$N(x_i, C_2) = N(x_i, 0) + \frac{1}{2}\ln[v_i(C_2)].$$

We can rewrite this, applying the above results to both sensitive and resistant cells, to express net growth rates in compartment i as functions of evofosfamide concentration C_2:

$$N_{X,i}(C_2) = N_{X,i}(0) + \frac{1}{2}\ln[v_i(C_2)],$$
$$N_{Y,i}(C_2) = N_{Y,i}(0) + \frac{1}{2}\ln[v_i(C_2)].$$

Note that in the above equations, $N_{X,i}(0)$ and $N_{Y,i}(0)$ are simply the net growth rates of sensitive and resistant cells in the absence of drug. In other words,

$$N_{X,i}(0) = \lambda^c_{X,i} - \mu^c_{X,i},$$
$$N_{Y,i}(0) = \lambda^c_{Y,i} - \mu^c_{Y,i},$$

where the superscript c denotes the control growth rates in the absence of drug, as defined in the first section of this document.

However, having explicitly defined functions for net growth rates of the sensitive and resistant cell populations is not quite enough; we must define their respective birth and death rates as well. Because evofosfamide is a cytotoxic drug, we assume it has no effect on birth rates, but rather increases death rates. Hence the birth rates of cells under any concentration of evofosfamide are equal to those in the absence of drug. Thus we define birth rates of sensitive and resistant cells in compartment i due to evofosfamide by

$$\lambda^2_{X,i}(t) = \lambda^c_{X,i},$$
$$\lambda^2_{Y,i}(t) = \lambda^c_{Y,i}.$$

Using the fact that net growth rates are differences between birth and death rates along with a series of substitutions of the above equations followed by algebraic simplification yields the following functions for death rates of sensitive and resistant cells in compartment i:

$$\mu^2_{X,i}(t) = \mu^c_{X,i} - \frac{1}{2}\ln[v_i(C_2(t))],$$
$$\mu^2_{Y,i}(t) = \mu^c_{Y,i} - \frac{1}{2}\ln[v_i(C_2(t))].$$

4. Derivation of evofosfamide plasma concentration function

Using pharmacokinetic data from a phase 1 clinical trial, we define a function $\rho_2(t)$ for plasma concentration over time after one dose of D_2 mg/m^2 evofosfamide. Unlike with erlotinib, we cannot simply approximate the plasma concentration of evofosfamide using only exponential decay because doing this would force the maximum concentration to be artificially high since evofosfamide decays so quickly with respect to time. This does not present a problem in the erlotinib plasma concentration function because the decay rate of erlotinib is much lower than that of evofosfamide. From available clinical data, we estimate the time at which maximum plasma concentration is achieved to be $t = \frac{1}{2}$ (30 minutes after the initial dose is administered). We approximate the plasma concentration for $t \geq \frac{1}{2}$ as exponential decay using $\rho_2(t) = c_2^{max} e^{-k_2(t-1/2)}$, where c_2^{max} is the maximum plasma concentration achieved after one dose and k_2 is the decay rate of evofosfamide. We acquire pairs of best-fit parameters for this function at a variety of doses between 7.5 and 940 mg/m^2 using the clinical trial data. Cubic functions are defined for both c_2^{max} and k_2 to describe the relationship between dose D_2 and maximum concentration and decay rate as follows:

$$c_2^{max}(D_2) = (2.008 \cdot 10^{-7})D_2^3 - 0.0003276D_2^2 + 0.1753D_2 - 12.54,$$
$$k_2(D_2) = (1.302 \cdot 10^{-7})D_2^3 - 0.0002043D_2^2 + 0.08873D_2 - 5.829.$$

To estimate the plasma concentration for $t < \frac{1}{2}$, we define a linear function connecting $(0, 0)$ with $\left(\frac{1}{2}, c_2^{max}(D_2)\right)$. We can now define evofosfamide plasma concentration after one dose as a function of time as follows:

$$\rho_2(t) = \begin{cases} 2c_2^{max}(D_2)t & t < \frac{1}{2} \\ c_2^{max}(D_2)e^{-k_2(D_2)(t-1/2)} & t \geq \frac{1}{2}, \end{cases}$$

where $c_2^{max}(D_2)$ and $k_2(D_2)$ are each defined as in the above equations.

5. Definition of one cycle of treatment in a combination dosing schedule

Here we describe in detail the process by which a single cycle of treatment in a combination dosing schedule is defined. We begin by calculating the length L of

the cycle, given n. Since n is the number of evofosfamide doses given in 3 weeks and there are 504 hours in 3 weeks, $L = 504/n$. Next we calculate the maximum tolerated dose of evofosfamide, given n, from the evofosfamide toxicity constraint curve. This dose of evofosfamide is administered at time $t = L - 24$ for Classes 1 and 2, and $t = L - 6$ for Class 3. The remaining time in the cycle, starting at $t = 0$ and going up to the time of the evofosfamide infusion, is then filled with the fixed erlotinib dosing schedule associated with the given optimization class. If necessary, doses of erlotinib are then removed, beginning with the dose immediately before evofosfamide is administered and working backward, until there is enough time between the last dose of erlotinib and the first dose of evofosfamide for the erlotinib plasma concentration to fall below 2.357 μM so as to not violate the combination toxicity constraint.

6. Explanation of the presence of two local minima in the optimization results

One interesting characteristic of the means of the sensitive and resistant cells in the optimization results is the presence of two local minima. Here we show that this phenomenon is due to the variability in timing between the last dose of erlotinib and the first dose of evofosfamide in each cycle.

To eliminate the variation in timing between doses, we re-define all dosing schedules in Class 1 so that either erlotinib or evofosfamide is administered every day with no additional doses in between. After calculating the length L of one cycle, we round $L - 24$ to the nearest 24 hours and define that to be the time of the evofosfamide infusion. Then, ignoring combination toxicity constraints solely for the purpose of this argument, we schedule erlotinib doses every 24 hours prior to the evofosfamide infusion. After re-defining dosing schedules in this manner, there no longer exists variability in the time between drug administrations since there is always a 24-hour waiting period between any two doses.

Originally, because the n evofosfamide doses were evenly divided into every three weeks, the end of treatment (at nine weeks) was also the end of a treatment cycle for every dosing schedule. However, that is no longer the case for these new daily dosing schedules since doses are forced to occur every 24 hours and only every 24 hours. Thus there are now two options for what to consider as the end of treatment. We may define the end of treatment to be exactly nine weeks from the treatment start date, but this introduces variability in the position within the cycle at which the resistance dynamics are calculated. The other option is to define the end of treatment to be the end of the cycle closest to nine weeks, but this will be at different times for different dosing schedules.

We examine both of these possibilities by re-calculating the means of the sensitive and resistant cells at both of these times for Class 1 under these new daily dosing schedules, shown in Fig. 2A and Fig. 2B, respectively. For the sake of comparison, the optimization results from the original dosing schedules in Class 1 are shown in blue. For each daily dosing schedule, the means of the sensitive and resistant cells are calculated at two different times: nine weeks from the start of treatment (yellow) and the end of the cycle closest to nine weeks after treatment begins (red). During the creation of the daily dosing schedules, the rounding of $L - 24$ to the nearest 24 hours leads to multiple dosing schedules with the same dose timing but different doses. To simplify the analysis, we only include one dosing schedule with

each particular schedule of doses in Fig. 2, chosen based on which n yields the value of $L - 24$ closest to the common rounded value.

Let us now compare the results from the original dosing schedules with those from the daily dosing schedules. The elimination of the variability in time between doses causes the first local minimum to become less pronounced for both the mean of the sensitive cells and the mean of the resistant cells, regardless of how the end of treatment is defined. Although the first local minimum still exists, its existence can now be easily explained in every case based on the different types of variation that have been introduced.

When the end of treatment is defined to be nine weeks after the treatment start date, this introduces variability in the relative position within the cycle at which the evolutionary dynamics are calculated. Specifically, the point at which this calculation occurs during treatment for $n = 3$ is at the very end of a cycle (after a large decline in the cancer cell population due to evofosfamide), whereas the point at which this calculation occurs for $n = 2$ and $n = 4$ is right before a dose of evofosfamide. This explains the existence of a local minimum at $n = 3$ for both means when the end of treatment is defined to be nine weeks after the start of treatment.

On the other hand, when the end of treatment is defined to be the end of a cycle, this introduces variability in the actual time at which the recurrence dynamics are calculated, and hence variability in the total treatment time. Specifically, the total treatment time for $n = 2$ is quite high relative to other values of n. Since the sensitive cell population is in a constant state of decline during treatment, this explains the existence of a local minimum at $n = 2$ for the mean of the sensitive cell population when the end of treatment is defined as the end of a cycle. Additionally, the total treatment time for $n = 3$ is quite low relative to other values of n. Since the resistant cell population is steadily growing by the end of treatment, this explains the existence of a local minimum at $n = 3$ for the mean of the resistant cells when the end of treatment is defined as the end of a cycle.

Note that for the mean of the sensitive cells, by following the curve for which the end of treatment is defined to be at nine weeks up to $n = 3$ and then the curve for which the end of treatment is defined to be the end of a cycle, the first local minimum is completely eliminated. This is a reasonable comparison to make because for $n = 3$, nine weeks after the start of treatment is the end of a treatment cycle. Together, all of the above evidence shows that the first local minimum is due to the variability in timing between the last dose of erlotinib and the first dose of evofosfamide in each cycle.

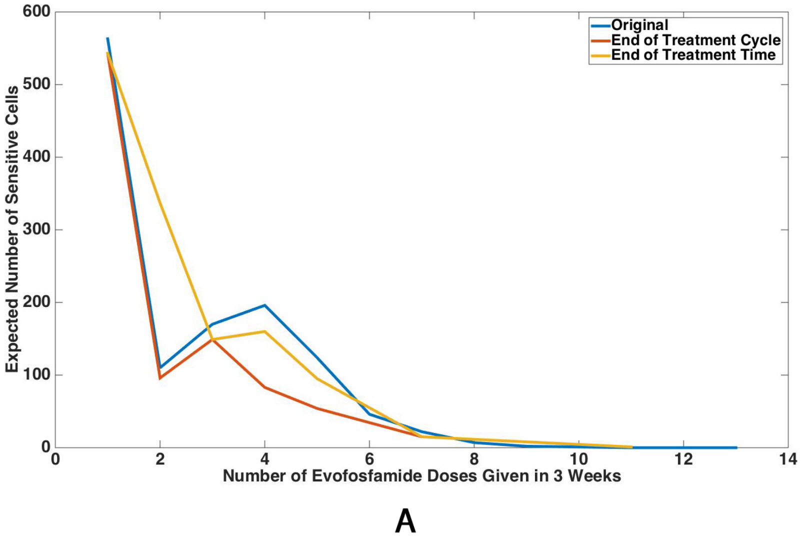

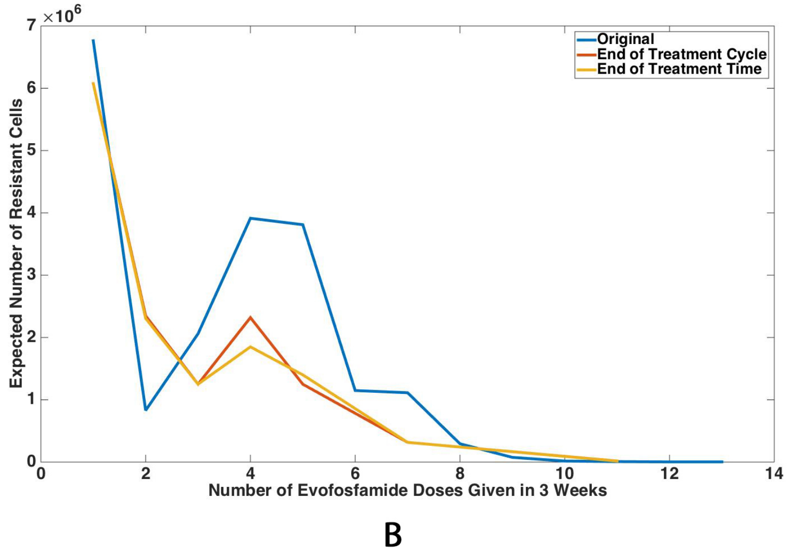

FIGURE 2. **A comparison of means of sensitive and resistant cells for dosing schedules with and without variation in amount of time between doses.** The means of the sensitive (A) and resistant (B) cell populations at the end of treatment for a tumor initially consisting of $1.6 \cdot 10^6$ sensitive cells are plotted for the original dosing schedules with variation in time between doses from Class 1 (blue) as well as the newly-defined daily dosing schedules, which eliminate this variation. For each daily dosing schedule, these means are calculated at two different times: nine weeks from the start of treatment (yellow) and the end of the cycle closest to nine weeks after treatment begins (red).

7. Impact of migration

To investigate the impact of migration on our model predictions, we relaxed the assumption that the evolutionary dynamics within each microenvironmental compartment are independent by introducing a migration term that allows cells to move between compartments. In addition to proliferating and dying, suppose cells in compartment i migrate to compartment j with rate $\rho(i,j)$. Assume that cells in compartment i can only migrate to compartment $i+1$ or $i-1$ at a given point in time. Then the means of the sensitive and resistant cells in each compartment are governed by a system of 64 ODE's. For $i = 1, 2, ..., 32$, we have

$$\begin{aligned}\frac{d}{dt}\mathbb{E}[X_i(t)] = &\Big(\lambda_{X,i}(t) - \mu_{X,i}(t) - \rho(i,i-1) - \rho(i,i+1)\Big)\mathbb{E}[X_i(t)] \\ &+ \rho(i-1,i)\mathbb{E}[X_{i-1}(t)] + \rho(i+1,i)\mathbb{E}[X_{i+1}(t)], \\ \frac{d}{dt}\mathbb{E}[Y_i(t)] = &\Big(\lambda_{Y,i}(t) - \mu_{Y,i}(t) - \rho(i,i-1) - \rho(i,i+1)\Big)\mathbb{E}[Y_i(t)] \\ &+ \lambda_{X,i}(t)u\mathbb{E}[X_i(t)] + \rho(i-1,i)\mathbb{E}[Y_{i-1}(t)] + \rho(i+1,i)\mathbb{E}[Y_{i+1}(t)].\end{aligned}$$

In the above equations, let $\rho(1,0) = \rho(0,1) = 0$ and $\rho(32,33) = \rho(33,32) = 0$ to account for the fact that there are only 32 compartments.

We defined the migration rate $\rho(i,j)$ using three different methods. Let the rates defined by these methods be denoted as $\rho_A(i,j)$, $\rho_B(i,j)$, and $\rho_C(i,j)$. For each compartment, by using the decay rate of oxygen away from the blood vessel together with the range of oxygen concentrations in that compartment, we estimated the range of distances from the blood vessel that correspond to the given compartment. We then used the minimum and maximum distances for each compartment to estimate a measurement of "width" - call this w_i for compartment i. To estimate w_i for the compartment furthest from the nearest blood vessel, we used the fact that the mean inter-capillary distance in tumor tissue is approximately 300 μm, so the point furthest from the nearest blood vessel in this compartment is at a distance of approximately 150 μm [2, 3]. We assume that cells travel at a rate of $r = 0.3$ μm/min [4]. The first method we used to define $\rho_A(i,j)$ assumes that cells only migrate toward the nearest blood vessel. So the migration rate is given by

$$\rho_A(i,j) = \begin{cases} \frac{r}{w_i} & i < j \\ 0 & i \geq j. \end{cases} \tag{4}$$

The second method we used assumes that cells only migrate away from the nearest blood vessel. Then the migration rate is given by

$$\rho_B(i,j) = \begin{cases} \frac{r}{w_i} & i > j \\ 0 & i \leq j. \end{cases} \tag{5}$$

For the third method, we allowed cells to migrate both toward and away from the nearest blood vessel with a constant migration rate of $\rho_C(i,j) = 0.1$. This is a reasonable estimate since the order of magnitude of r is -1 and for most compartments, w_i has order of magnitude 0.

To examine the impact of migration on the mean tumor size, we calculated mean tumor size over time for two different combination dosing schedules using migration defined in each of the three methods described above as well as without

migration. Fig. 3A shows the results of this calculation for a dosing schedule that combines a standard 150 mg daily erlotinib schedule with a standard 575 mg/m^2 weekly evofosfamide schedule. Fig. 3B shows the results for the combination dosing schedule given by $n = 14$ in Class 3, which was found to be the optimal dosing schedule to minimize mean tumor size in the previous section.

We find that migration does have an impact on our model predictions with regards to mean tumor size. Since the majority of the cancer cells are initially in hypoxic regions of the tumor microenvironment, allowing migration between compartments governed by a constant migration rate leads to a more well-mixed population. Therefore, with a constant migration rate incorporated into the model, erlotinib has more of an effect on the population while evofosfamide has less of an effect since the cells are more evenly distributed throughout the tumor microenvironment. This can be seen in Fig. 3A and Fig. 3B. This same phenomenon can be observed in the case where cells only migrate toward the blood vessel, although it is more pronounced in this situation since cells are only allowed to move away from the hypoxic regions of the tumor microenvironment. On the other hand, by incorporating migration such that cells may only move away from the blood vessel, the observed shift in mean tumor size is likely due to a decreased response to erlotinib and an increased response to evofosfamide due to the fact that the cancer cells are even more concentrated in hypoxic regions.

These results indicate that unidirectional migration of cell populations either towards or away from blood vessels have more of an impact on the model-predicted mean tumor evolution than bidirectional migration rates. However, the migration rates chosen here fell on the higher end of literature-reported values, and it is likely that migration patterns found in vivo are multidirectional, heterogeneous and dependent upon cellular density in local and surrounding compartments. In addition, it is possible that sensitive and resistant cells may have different migration speeds or capabilities. To better quantify the impact of migration on the evolutionary dynamics of the tumor population, further experimental investigation is needed to quantify migration rates and develop a more refined mathematical model of local migration between compartments.

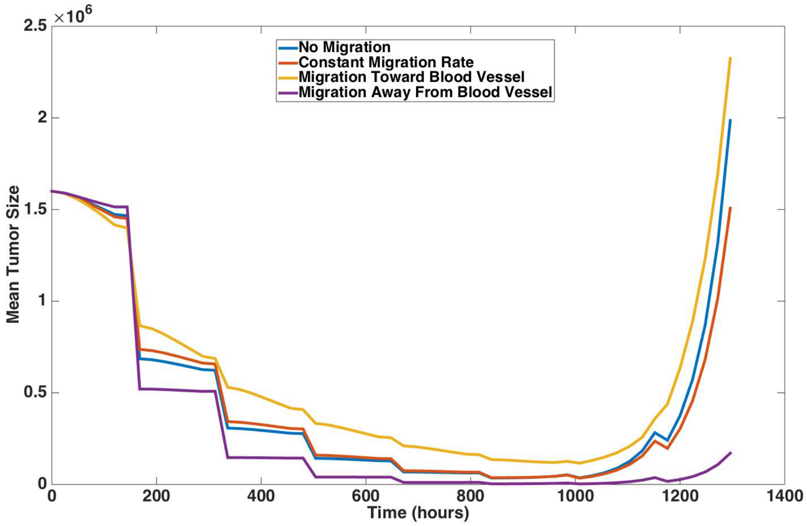

A

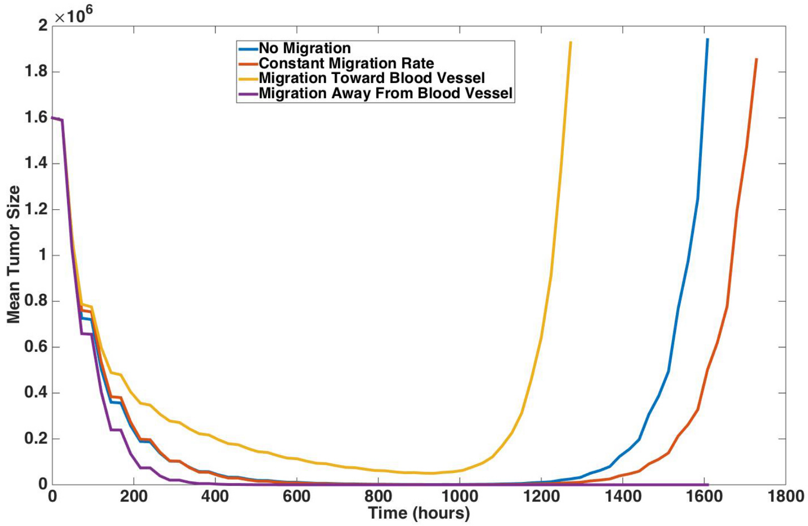

B

FIGURE 3. **A comparison of mean tumor size with and without migration.** Mean tumor size over time is plotted for two different combination dosing schedules in (A) and (B) using no migration (blue curves), a constant migration rate (red curves), a migration rate in which cells can only move toward the nearest blood vessel (yellow curves), and a migration rate in which cells can only move away from the nearest blood vessel (purple curves).

8. Supplementary matrices

Here we show the matrices SB, SD, RB, and RD used to calculate the birth and death rate functions of erlotinib-sensitive and erlotinib-resistant cells, defined in the first section of this document.

$$SB = \begin{bmatrix}
1.1053393e{-}02 & 4.7156432e{-}04 & 7.7651360e{-}03 \\
5.4991358e{-}04 & 1.1535643e{-}05 & 5.9142865e{-}04 \\
5.4989360e{-}04 & 1.1528540e{-}05 & 5.9146688e{-}04 \\
5.4989360e{-}04 & 1.1528540e{-}05 & 5.9146688e{-}04 \\
5.4989360e{-}04 & 1.1528540e{-}05 & 5.9146688e{-}04 \\
5.4989360e{-}04 & 1.1528540e{-}05 & 5.9146688e{-}04 \\
5.4989360e{-}04 & 1.1528540e{-}05 & 5.9146688e{-}04 \\
5.4989360e{-}04 & 1.1528540e{-}05 & 5.9146688e{-}04 \\
5.4989360e{-}04 & 1.1528540e{-}05 & 5.9146688e{-}04 \\
5.4989360e{-}04 & 1.1528540e{-}05 & 5.9146688e{-}04 \\
5.4989360e{-}04 & 1.1528540e{-}05 & 5.9146688e{-}04 \\
5.4989360e{-}04 & 1.1528540e{-}05 & 5.9146688e{-}04 \\
5.4989360e{-}04 & 1.1528540e{-}05 & 5.9146688e{-}04 \\
5.4989360e{-}04 & 1.1528540e{-}05 & 5.9146688e{-}04 \\
5.4989360e{-}04 & 1.1528540e{-}05 & 5.9146688e{-}04 \\
5.4989360e{-}04 & 1.1528540e{-}05 & 5.9146688e{-}04 \\
5.4989360e{-}04 & 1.1528540e{-}05 & 5.9146688e{-}04 \\
5.4989360e{-}04 & 1.1528540e{-}05 & 5.9146688e{-}04 \\
5.4989360e{-}04 & 1.1528540e{-}05 & 5.9146688e{-}04 \\
5.4989360e{-}04 & 1.1528540e{-}05 & 5.9146688e{-}04 \\
5.4989360e{-}04 & 1.1528540e{-}05 & 5.9146688e{-}04 \\
5.4989360e{-}04 & 1.1528540e{-}05 & 5.9146688e{-}04 \\
5.4989360e{-}04 & 1.1528540e{-}05 & 5.9146688e{-}04 \\
5.4989360e{-}04 & 1.1528540e{-}05 & 5.9146688e{-}04 \\
5.4989360e{-}04 & 1.1528540e{-}05 & 5.9146688e{-}04 \\
5.4989360e{-}04 & 1.1528540e{-}05 & 5.9146688e{-}04 \\
5.4989360e{-}04 & 1.1528540e{-}05 & 5.9146688e{-}04 \\
5.4989360e{-}04 & 1.1528540e{-}05 & 5.9146688e{-}04 \\
5.4989360e{-}04 & 1.1528540e{-}05 & 5.9146688e{-}04 \\
5.4989360e{-}04 & 1.1528540e{-}05 & 5.9146688e{-}04 \\
5.4989360e{-}04 & 1.1528540e{-}05 & 5.9146688e{-}04 \\
5.4989360e{-}04 & 1.1528540e{-}05 & 5.9146688e{-}04 \\
5.4989360e{-}04 & 1.1528540e{-}05 & 5.9146688e{-}04 \\
5.4989360e{-}04 & 1.1528540e{-}05 & 5.9146688e{-}04 \\
5.4989360e{-}04 & 1.1528540e{-}05 & 5.9146688e{-}04 \\
5.4989360e{-}04 & 1.1528540e{-}05 & 5.9146688e{-}04 \\
5.4989360e{-}04 & 1.1528540e{-}05 & 5.9146688e{-}04 \\
5.4989360e{-}04 & 1.1528540e{-}05 & 5.9146688e{-}04 \\
5.4989360e{-}04 & 1.1528540e{-}05 & 5.9146688e{-}04 \\
5.4989360e{-}04 & 1.1528540e{-}05 & 5.9146688e{-}04 \\
5.4989360e{-}04 & 1.1528540e{-}05 & 5.9146688e{-}04 \\
5.4989360e{-}04 & 1.1528540e{-}05 & 5.9146688e{-}04 \\
5.4989360e{-}04 & 1.1528540e{-}05 & 5.9146688e{-}04 \\
5.4989360e{-}04 & 1.1528540e{-}05 & 5.9146688e{-}04 \\
5.4989360e{-}04 & 1.1528540e{-}05 & 5.9146688e{-}04 \\
5.4989360e{-}04 & 1.1528540e{-}05 & 5.9146688e{-}04 \\
5.4989360e{-}04 & 1.1528540e{-}05 & 5.9146688e{-}04 \\
5.4989360e{-}04 & 1.1528540e{-}05 & 5.9146688e{-}04 \\
5.4989360e{-}04 & 1.1528540e{-}05 & 5.9146688e{-}04 \\
5.4989360e{-}04 & 1.1528540e{-}05 & 5.9146688e{-}04 \\
5.4989360e{-}04 & 1.1528540e{-}05 & 5.9146688e{-}04 \\
5.4989360e{-}04 & 1.1528540e{-}05 & 5.9146688e{-}04 \\
5.4989360e{-}04 & 1.1528540e{-}05 & 5.9146688e{-}04 \\
5.4989360e{-}04 & 1.1528540e{-}05 & 5.9146688e{-}04 \\
5.4989360e{-}04 & 1.1528540e{-}05 & 5.9146688e{-}04 \\
5.4989360e{-}04 & 1.1528540e{-}05 & 5.9146688e{-}04 \\
5.4989360e{-}04 & 1.1528540e{-}05 & 5.9146688e{-}04 \\
5.4989360e{-}04 & 1.1528540e{-}05 & 5.9146688e{-}04 \\
5.4989360e{-}04 & 1.1528540e{-}05 & 5.9146688e{-}04 \\
5.4989360e{-}04 & 1.1528540e{-}05 & 5.9146688e{-}04 \\
5.4989360e{-}04 & 1.1528540e{-}05 & 5.9146688e{-}04 \\
5.4989360e{-}04 & 1.1528540e{-}05 & 5.9146688e{-}04 \\
5.4989360e{-}04 & 1.1528540e{-}05 & 5.9146688e{-}04 \\
5.4989360e{-}04 & 1.1528540e{-}05 & 5.9146688e{-}04 \\
5.4989360e{-}04 & 1.1528540e{-}05 & 5.9146688e{-}04 \\
5.4989360e{-}04 & 1.1528540e{-}05 & 5.9146688e{-}04 \\
5.4989360e{-}04 & 1.1528540e{-}05 & 5.9146688e{-}04 \\
5.4989360e{-}04 & 1.1528540e{-}05 & 5.9146688e{-}04 \\
5.4989360e{-}04 & 1.1528540e{-}05 & 5.9146688e{-}04 \\
5.4989360e{-}04 & 1.1528540e{-}05 & 5.9146688e{-}04 \\
5.4989360e{-}04 & 1.1528540e{-}05 & 5.9146688e{-}04 \\
5.4989360e{-}04 & 1.1528540e{-}05 & 5.9146688e{-}04 \\
5.4989360e{-}04 & 1.1528540e{-}05 & 5.9146688e{-}04 \\
5.4989360e{-}04 & 1.1528540e{-}05 & 5.9146688e{-}04 \\
5.4989360e{-}04 & 1.1528540e{-}05 & 5.9146688e{-}04 \\
5.4989360e{-}04 & 1.1528540e{-}05 & 5.9146688e{-}04 \\
5.4989360e{-}04 & 1.1528540e{-}05 & 5.9146688e{-}04 \\
5.4989360e{-}04 & 1.1528540e{-}05 & 5.9146688e{-}04 \\
5.4989360e{-}04 & 1.1528540e{-}05 & 5.9146688e{-}04 \\
5.4989360e{-}04 & 1.1528540e{-}05 & 5.9146688e{-}04 \\
5.4989360e{-}04 & 1.1528540e{-}05 & 5.9146688e{-}04 \\
5.4989360e{-}04 & 1.1528540e{-}05 & 5.9146688e{-}04 \\
5.4989360e{-}04 & 1.1528540e{-}05 & 5.9146688e{-}04 \\
5.4989360e{-}04 & 1.1528540e{-}05 & 5.9146688e{-}04 \\
5.4989360e{-}04 & 1.1528540e{-}05 & 5.9146688e{-}04 \\
5.4989360e{-}04 & 1.1528540e{-}05 & 5.9146688e{-}04 \\
5.4989360e{-}04 & 1.1528540e{-}05 & 5.9146688e{-}04 \\
5.4989360e{-}04 & 1.1528540e{-}05 & 5.9146688e{-}04 \\
5.4989360e{-}04 & 1.1528540e{-}05 & 5.9146688e{-}04 \\
5.4989360e{-}04 & 1.1528540e{-}05 & 5.9146688e{-}04 \\
5.4989360e{-}04 & 1.1528540e{-}05 & 5.9146688e{-}04 \\
5.4989360e{-}04 & 1.1528540e{-}05 & 5.9146688e{-}04 \\
5.4989360e{-}04 & 1.1528540e{-}05 & 5.9146688e{-}04 \\
5.4989360e{-}04 & 1.1528540e{-}05 & 5.9146688e{-}04 \\
5.4989360e{-}04 & 1.1528540e{-}05 & 5.9146688e{-}04 \\
5.4989360e{-}04 & 1.1528540e{-}05 & 5.9146688e{-}04 \\
5.4989360e{-}04 & 1.1528540e{-}05 & 5.9146688e{-}04 \\
5.4989360e{-}04 & 1.1528540e{-}05 & 5.9146688e{-}04 \\
5.4989360e{-}04 & 1.1528540e{-}05 & 5.9146688e{-}04 \\
5.4989360e{-}04 & 1.1528540e{-}05 & 5.9146688e{-}04
\end{bmatrix}$$

$$SD = \begin{bmatrix}
1.4792648e{-03} & -2.7085135e{-05} & -4.5609723e{-04} \\
1.5481068e{-03} & -2.5930657e{-05} & -4.5845635e{-04} \\
1.6169489e{-03} & -2.4776179e{-05} & -4.6081546e{-04} \\
1.6857909e{-03} & -2.3621701e{-05} & -4.6317457e{-04} \\
1.7546330e{-03} & -2.2467222e{-05} & -4.6553369e{-04} \\
1.8234750e{-03} & -2.1312744e{-05} & -4.6789280e{-04} \\
1.8923170e{-03} & -2.0158266e{-05} & -4.7025192e{-04} \\
1.9611591e{-03} & -1.9003788e{-05} & -4.7261103e{-04} \\
2.0300011e{-03} & -1.7849310e{-05} & -4.7497014e{-04} \\
2.0988432e{-03} & -1.6694831e{-05} & -4.7732926e{-04} \\
2.1676852e{-03} & -1.5540353e{-05} & -4.7968837e{-04} \\
2.2365272e{-03} & -1.4385875e{-05} & -4.8204748e{-04} \\
2.3053693e{-03} & -1.3231397e{-05} & -4.8440660e{-04} \\
2.3742113e{-03} & -1.2076919e{-05} & -4.8676571e{-04} \\
2.4430534e{-03} & -1.0922440e{-05} & -4.8912483e{-04} \\
2.5118954e{-03} & -9.7679622e{-06} & -4.9148394e{-04} \\
2.5807374e{-03} & -8.6134840e{-06} & -4.9384305e{-04} \\
2.6495795e{-03} & -7.4590058e{-06} & -4.9620217e{-04} \\
2.7184215e{-03} & -6.3045276e{-06} & -4.9856128e{-04} \\
2.7872635e{-03} & -5.1500494e{-06} & -5.0092040e{-04} \\
2.8561056e{-03} & -3.9955712e{-06} & -5.0327951e{-04} \\
2.9249476e{-03} & -2.8410930e{-06} & -5.0563862e{-04} \\
2.9937897e{-03} & -1.6866148e{-06} & -5.0799774e{-04} \\
3.0626317e{-03} & -5.3213659e{-07} & -5.1035685e{-04} \\
3.1314737e{-03} & 6.2234162e{-07} & -5.1271597e{-04} \\
3.2003158e{-03} & 1.7768198e{-06} & -5.1507508e{-04} \\
3.2691578e{-03} & 2.9312980e{-06} & -5.1743419e{-04} \\
3.3379999e{-03} & 4.0857762e{-06} & -5.1979331e{-04} \\
3.4068419e{-03} & 5.2402544e{-06} & -5.2215242e{-04} \\
3.4756839e{-03} & 6.3947326e{-06} & -5.2451154e{-04} \\
3.5445260e{-03} & 7.5492109e{-06} & -5.2687065e{-04} \\
3.6133680e{-03} & 8.7036891e{-06} & -5.2922976e{-04} \\
3.6822101e{-03} & 9.8581673e{-06} & -5.3158888e{-04} \\
3.7510521e{-03} & 1.1012645e{-05} & -5.3394799e{-04} \\
3.8198941e{-03} & 1.2167124e{-05} & -5.3630711e{-04} \\
3.8887362e{-03} & 1.3321602e{-05} & -5.3866622e{-04} \\
3.9575782e{-03} & 1.4476080e{-05} & -5.4102533e{-04} \\
4.0264203e{-03} & 1.5630558e{-05} & -5.4338445e{-04} \\
4.0952623e{-03} & 1.6785036e{-05} & -5.4574356e{-04} \\
4.1641043e{-03} & 1.7939515e{-05} & -5.4810267e{-04} \\
4.2329464e{-03} & 1.9093993e{-05} & -5.5046179e{-04} \\
4.3017884e{-03} & 2.0248471e{-05} & -5.5282090e{-04} \\
4.3706305e{-03} & 2.1402949e{-05} & -5.5518002e{-04} \\
4.4394725e{-03} & 2.2557428e{-05} & -5.5753913e{-04} \\
4.5083145e{-03} & 2.3711906e{-05} & -5.5989824e{-04} \\
4.5771566e{-03} & 2.4866384e{-05} & -5.6225736e{-04} \\
4.6459986e{-03} & 2.6020862e{-05} & -5.6461647e{-04} \\
4.7148406e{-03} & 2.7175340e{-05} & -5.6697559e{-04} \\
4.7836827e{-03} & 2.8329819e{-05} & -5.6933470e{-04} \\
4.8525247e{-03} & 2.9484297e{-05} & -5.7169381e{-04} \\
4.9213668e{-03} & 3.0638775e{-05} & -5.7405293e{-04} \\
4.9902088e{-03} & 3.1793253e{-05} & -5.7641204e{-04} \\
5.0590508e{-03} & 3.2947731e{-05} & -5.7877116e{-04} \\
5.1278929e{-03} & 3.4102210e{-05} & -5.8113027e{-04} \\
5.1967349e{-03} & 3.5256688e{-05} & -5.8348938e{-04} \\
5.2655770e{-03} & 3.6411166e{-05} & -5.8584850e{-04} \\
5.3344190e{-03} & 3.7565644e{-05} & -5.8820761e{-04} \\
5.4032610e{-03} & 3.8720122e{-05} & -5.9056673e{-04} \\
5.4721031e{-03} & 3.9874601e{-05} & -5.9292584e{-04} \\
5.5409451e{-03} & 4.1029079e{-05} & -5.9528495e{-04} \\
5.6097872e{-03} & 4.2183557e{-05} & -5.9764407e{-04} \\
5.6786292e{-03} & 4.3338035e{-05} & -6.0000318e{-04} \\
5.7474712e{-03} & 4.4492513e{-05} & -6.0236230e{-04} \\
5.8163133e{-03} & 4.5646992e{-05} & -6.0472141e{-04} \\
5.8851553e{-03} & 4.6801470e{-05} & -6.0708052e{-04} \\
5.9539974e{-03} & 4.7955948e{-05} & -6.0943964e{-04} \\
6.0228394e{-03} & 4.9110426e{-05} & -6.1179875e{-04} \\
6.0916814e{-03} & 5.0264904e{-05} & -6.1415786e{-04} \\
6.1605235e{-03} & 5.1419383e{-05} & -6.1651698e{-04} \\
6.2293655e{-03} & 5.2573861e{-05} & -6.1887609e{-04} \\
6.2982076e{-03} & 5.3728339e{-05} & -6.2123521e{-04} \\
6.3670496e{-03} & 5.4882817e{-05} & -6.2359432e{-04} \\
6.4358916e{-03} & 5.6037295e{-05} & -6.2595343e{-04} \\
6.5047337e{-03} & 5.7191774e{-05} & -6.2831255e{-04} \\
6.5735757e{-03} & 5.8346252e{-05} & -6.3067166e{-04} \\
6.6424177e{-03} & 5.9500730e{-05} & -6.3303078e{-04} \\
6.7112598e{-03} & 6.0655208e{-05} & -6.3538989e{-04} \\
6.7801018e{-03} & 6.1809687e{-05} & -6.3774900e{-04} \\
6.8489439e{-03} & 6.2964165e{-05} & -6.4010812e{-04} \\
6.9177859e{-03} & 6.4118643e{-05} & -6.4246723e{-04} \\
6.9866279e{-03} & 6.5273121e{-05} & -6.4482635e{-04} \\
7.0554700e{-03} & 6.6427599e{-05} & -6.4718546e{-04} \\
7.1243120e{-03} & 6.7582078e{-05} & -6.4954457e{-04} \\
7.1931541e{-03} & 6.8736556e{-05} & -6.5190369e{-04} \\
7.2619961e{-03} & 6.9891034e{-05} & -6.5426280e{-04} \\
7.3308381e{-03} & 7.1045512e{-05} & -6.5662192e{-04} \\
7.3996802e{-03} & 7.2199990e{-05} & -6.5898103e{-04} \\
7.4685222e{-03} & 7.3354469e{-05} & -6.6134014e{-04} \\
7.5373643e{-03} & 7.4508947e{-05} & -6.6369926e{-04} \\
7.6062063e{-03} & 7.5663425e{-05} & -6.6605837e{-04} \\
7.6750483e{-03} & 7.6817903e{-05} & -6.6841749e{-04} \\
7.7438904e{-03} & 7.7972381e{-05} & -6.7077660e{-04} \\
7.8127324e{-03} & 7.9126860e{-05} & -6.7313571e{-04} \\
7.8815745e{-03} & 8.0281338e{-05} & -6.7549483e{-04} \\
7.9504165e{-03} & 8.1435816e{-05} & -6.7785394e{-04} \\
8.0192585e{-03} & 8.2590294e{-05} & -6.8021306e{-04} \\
8.0881006e{-03} & 8.3744772e{-05} & -6.8257217e{-04} \\
8.1569426e{-03} & 8.4899251e{-05} & -6.8493128e{-04} \\
8.2257846e{-03} & 8.6053729e{-05} & -6.8729040e{-04} \\
8.2946267e{-03} & 8.7208207e{-05} & -6.8964951e{-04} \\
8.3634687e{-03} & 8.8362685e{-05} & -6.9200862e{-04}
\end{bmatrix}$$

$$
RB = \begin{bmatrix}
1.0085430e{-02} & 1.0771499e{-04} & 8.7460853e{-03} \\
1.0022742e{-02} & 1.0536662e{-04} & 8.6793665e{-03} \\
9.9600535e{-03} & 1.0301824e{-04} & 8.6126477e{-03} \\
9.8973651e{-03} & 1.0066987e{-04} & 8.5459288e{-03} \\
9.8346767e{-03} & 9.8321502e{-05} & 8.4792100e{-03} \\
9.7719883e{-03} & 9.5973131e{-05} & 8.4124912e{-03} \\
9.7092999e{-03} & 9.3624760e{-05} & 8.3457724e{-03} \\
9.6466115e{-03} & 9.1276388e{-05} & 8.2790536e{-03} \\
9.5839231e{-03} & 8.8928017e{-05} & 8.2123348e{-03} \\
9.5212347e{-03} & 8.6579646e{-05} & 8.1456160e{-03} \\
9.4585463e{-03} & 8.4231275e{-05} & 8.0788971e{-03} \\
9.3958579e{-03} & 8.1882904e{-05} & 8.0121783e{-03} \\
9.3331695e{-03} & 7.9534532e{-05} & 7.9454595e{-03} \\
9.2704811e{-03} & 7.7186161e{-05} & 7.8787407e{-03} \\
9.2077927e{-03} & 7.4837790e{-05} & 7.8120219e{-03} \\
9.1451043e{-03} & 7.2489419e{-05} & 7.7453031e{-03} \\
9.0824159e{-03} & 7.0141048e{-05} & 7.6785843e{-03} \\
9.0197275e{-03} & 6.7792676e{-05} & 7.6118654e{-03} \\
8.9570391e{-03} & 6.5444305e{-05} & 7.5451466e{-03} \\
8.8943507e{-03} & 6.3095934e{-05} & 7.4784278e{-03} \\
8.8316623e{-03} & 6.0747563e{-05} & 7.4117090e{-03} \\
8.7689739e{-03} & 5.8399191e{-05} & 7.3449902e{-03} \\
8.7062855e{-03} & 5.6050820e{-05} & 7.2782714e{-03} \\
8.6435971e{-03} & 5.3702449e{-05} & 7.2115526e{-03} \\
8.5809087e{-03} & 5.1354078e{-05} & 7.1448337e{-03} \\
8.5182203e{-03} & 4.9005707e{-05} & 7.0781149e{-03} \\
8.4555319e{-03} & 4.6657335e{-05} & 7.0113961e{-03} \\
8.3928435e{-03} & 4.4308964e{-05} & 6.9446773e{-03} \\
8.3301551e{-03} & 4.1960593e{-05} & 6.8779585e{-03} \\
8.2674667e{-03} & 3.9612222e{-05} & 6.8112397e{-03} \\
8.2047783e{-03} & 3.7263850e{-05} & 6.7445209e{-03} \\
8.1420899e{-03} & 3.4915479e{-05} & 6.6778020e{-03} \\
8.0794015e{-03} & 3.2567108e{-05} & 6.6110832e{-03} \\
8.0167131e{-03} & 3.0218737e{-05} & 6.5443644e{-03} \\
7.9540247e{-03} & 2.7870366e{-05} & 6.4776456e{-03} \\
7.8913363e{-03} & 2.5521994e{-05} & 6.4109268e{-03} \\
7.8286479e{-03} & 2.3173623e{-05} & 6.3442080e{-03} \\
7.7659595e{-03} & 2.0825252e{-05} & 6.2774892e{-03} \\
7.7032711e{-03} & 1.8476881e{-05} & 6.2107703e{-03} \\
7.6405827e{-03} & 1.6128510e{-05} & 6.1440515e{-03} \\
7.5778943e{-03} & 1.3780138e{-05} & 6.0773327e{-03} \\
7.5152059e{-03} & 1.1431767e{-05} & 6.0106139e{-03} \\
7.4525175e{-03} & 9.0833959e{-06} & 5.9438951e{-03} \\
7.3898291e{-03} & 6.7350247e{-06} & 5.8771763e{-03} \\
7.3271407e{-03} & 4.3866534e{-06} & 5.8104575e{-03} \\
7.2644523e{-03} & 2.0382822e{-06} & 5.7437387e{-03} \\
7.2017639e{-03} & -3.1008899e{-07} & 5.6770198e{-03} \\
7.1390755e{-03} & -2.6584602e{-06} & 5.6103010e{-03} \\
7.0763871e{-03} & -5.0068314e{-06} & 5.5435822e{-03} \\
7.0136987e{-03} & -7.3552026e{-06} & 5.4768634e{-03} \\
6.9510103e{-03} & -9.7035739e{-06} & 5.4101446e{-03} \\
6.8883219e{-03} & -1.2051945e{-05} & 5.3434258e{-03} \\
6.8256335e{-03} & -1.4400316e{-05} & 5.2767070e{-03} \\
6.7629451e{-03} & -1.6748688e{-05} & 5.2099881e{-03} \\
6.7002567e{-03} & -1.9097059e{-05} & 5.1432693e{-03} \\
6.6375683e{-03} & -2.1445430e{-05} & 5.0765505e{-03} \\
6.5748799e{-03} & -2.3793801e{-05} & 5.0098317e{-03} \\
6.5121915e{-03} & -2.6142172e{-05} & 4.9431129e{-03} \\
6.4495031e{-03} & -2.8490544e{-05} & 4.8763941e{-03} \\
6.3868147e{-03} & -3.0838915e{-05} & 4.8096753e{-03} \\
6.3241263e{-03} & -3.3187286e{-05} & 4.7429564e{-03} \\
6.2614379e{-03} & -3.5535657e{-05} & 4.6762376e{-03} \\
6.1987495e{-03} & -3.7884028e{-05} & 4.6095188e{-03} \\
6.1360611e{-03} & -4.0232400e{-05} & 4.5428000e{-03} \\
6.0733727e{-03} & -4.2580771e{-05} & 4.4760812e{-03} \\
6.0106843e{-03} & -4.4929142e{-05} & 4.4093624e{-03} \\
5.9479959e{-03} & -4.7277513e{-05} & 4.3426436e{-03} \\
5.8853075e{-03} & -4.9625885e{-05} & 4.2759247e{-03} \\
5.8226191e{-03} & -5.1974256e{-05} & 4.2092059e{-03} \\
5.7599307e{-03} & -5.4322627e{-05} & 4.1424871e{-03} \\
5.6972423e{-03} & -5.6670998e{-05} & 4.0757683e{-03} \\
5.6345539e{-03} & -5.9019369e{-05} & 4.0090495e{-03} \\
5.5718655e{-03} & -6.1367741e{-05} & 3.9423307e{-03} \\
5.5091771e{-03} & -6.3716112e{-05} & 3.8756119e{-03} \\
5.4464887e{-03} & -6.6064483e{-05} & 3.8088930e{-03} \\
5.3838003e{-03} & -6.8412854e{-05} & 3.7421742e{-03} \\
5.3211119e{-03} & -7.0761226e{-05} & 3.6754554e{-03} \\
5.2584235e{-03} & -7.3109597e{-05} & 3.6087366e{-03} \\
5.1957351e{-03} & -7.5457968e{-05} & 3.5420178e{-03} \\
5.1330467e{-03} & -7.7806339e{-05} & 3.4752990e{-03} \\
5.0703583e{-03} & -8.0154710e{-05} & 3.4085802e{-03} \\
5.0076699e{-03} & -8.2503082e{-05} & 3.3418613e{-03} \\
4.9449815e{-03} & -8.4851453e{-05} & 3.2751425e{-03} \\
4.8822931e{-03} & -8.7199824e{-05} & 3.2084237e{-03} \\
4.8196047e{-03} & -8.9548195e{-05} & 3.1417049e{-03} \\
4.7569163e{-03} & -9.1896566e{-05} & 3.0749861e{-03} \\
4.6942279e{-03} & -9.4244938e{-05} & 3.0082673e{-03} \\
4.6315395e{-03} & -9.6593309e{-05} & 2.9415485e{-03} \\
4.5688511e{-03} & -9.8941680e{-05} & 2.8748296e{-03} \\
4.5061627e{-03} & -1.0129005e{-04} & 2.8081108e{-03} \\
4.4434743e{-03} & -1.0363842e{-04} & 2.7413920e{-03} \\
4.3807859e{-03} & -1.0598679e{-04} & 2.6746732e{-03} \\
4.3180975e{-03} & -1.0833516e{-04} & 2.6079544e{-03} \\
4.2554091e{-03} & -1.1068354e{-04} & 2.5412356e{-03} \\
4.1927207e{-03} & -1.1303191e{-04} & 2.4745168e{-03} \\
4.1300323e{-03} & -1.1538028e{-04} & 2.4077979e{-03} \\
4.0673439e{-03} & -1.1772865e{-04} & 2.3410791e{-03} \\
4.0046555e{-03} & -1.2007702e{-04} & 2.2743603e{-03} \\
3.9419671e{-03} & -1.2242539e{-04} & 2.2076415e{-03} \\
3.8792787e{-03} & -1.2477376e{-04} & 2.1409227e{-03} \\
3.8165903e{-03} & -1.2712213e{-04} & 2.0742039e{-03}
\end{bmatrix}
$$

$$RD = \begin{bmatrix}
2.5417382e-04 & 3.0307471e-05 & -1.5361632e-04 \\
2.7572098e-04 & 2.9925193e-05 & -1.5577968e-04 \\
2.9726815e-04 & 2.9542914e-05 & -1.5794303e-04 \\
3.1881531e-04 & 2.9160635e-05 & -1.6010639e-04 \\
3.4036248e-04 & 2.8778356e-05 & -1.6226974e-04 \\
3.6190964e-04 & 2.8396077e-05 & -1.6443310e-04 \\
3.8345681e-04 & 2.8013799e-05 & -1.6659645e-04 \\
4.0500398e-04 & 2.7631520e-05 & -1.6875981e-04 \\
4.2655114e-04 & 2.7249241e-05 & -1.7092316e-04 \\
4.4809831e-04 & 2.6866962e-05 & -1.7308652e-04 \\
4.6964547e-04 & 2.6484683e-05 & -1.7524987e-04 \\
4.9119264e-04 & 2.6102404e-05 & -1.7741323e-04 \\
5.1273980e-04 & 2.5720126e-05 & -1.7957658e-04 \\
5.3428697e-04 & 2.5337847e-05 & -1.8173994e-04 \\
5.5583413e-04 & 2.4955568e-05 & -1.8390329e-04 \\
5.7738130e-04 & 2.4573289e-05 & -1.8606665e-04 \\
5.9892847e-04 & 2.4191010e-05 & -1.8823000e-04 \\
6.2047563e-04 & 2.3808732e-05 & -1.9039336e-04 \\
6.4202280e-04 & 2.3426453e-05 & -1.9255671e-04 \\
6.6356996e-04 & 2.3044174e-05 & -1.9472007e-04 \\
6.8511713e-04 & 2.2661895e-05 & -1.9688342e-04 \\
7.0666429e-04 & 2.2279616e-05 & -1.9904678e-04 \\
7.2821146e-04 & 2.1897338e-05 & -2.0121013e-04 \\
7.4975862e-04 & 2.1515059e-05 & -2.0337349e-04 \\
7.7130579e-04 & 2.1132780e-05 & -2.0553684e-04 \\
7.9285296e-04 & 2.0750501e-05 & -2.0770020e-04 \\
8.1440012e-04 & 2.0368222e-05 & -2.0986355e-04 \\
8.3594729e-04 & 1.9985943e-05 & -2.1202691e-04 \\
8.5749445e-04 & 1.9603665e-05 & -2.1419026e-04 \\
8.7904162e-04 & 1.9221386e-05 & -2.1635362e-04 \\
9.0058878e-04 & 1.8839107e-05 & -2.1851697e-04 \\
9.2213595e-04 & 1.8456828e-05 & -2.2068033e-04 \\
9.4368311e-04 & 1.8074549e-05 & -2.2284368e-04 \\
9.6523028e-04 & 1.7692271e-05 & -2.2500704e-04 \\
9.8677745e-04 & 1.7309992e-05 & -2.2717039e-04 \\
1.0083246e-03 & 1.6927713e-05 & -2.2933375e-04 \\
1.0298718e-03 & 1.6545434e-05 & -2.3149710e-04 \\
1.0514189e-03 & 1.6163155e-05 & -2.3366046e-04 \\
1.0729661e-03 & 1.5780876e-05 & -2.3582381e-04 \\
1.0945133e-03 & 1.5398598e-05 & -2.3798717e-04 \\
1.1160604e-03 & 1.5016319e-05 & -2.4015052e-04 \\
1.1376076e-03 & 1.4634040e-05 & -2.4231388e-04 \\
1.1591548e-03 & 1.4251761e-05 & -2.4447723e-04 \\
1.1807019e-03 & 1.3869482e-05 & -2.4664059e-04 \\
1.2022491e-03 & 1.3487204e-05 & -2.4880395e-04 \\
1.2237963e-03 & 1.3104925e-05 & -2.5096730e-04 \\
1.2453434e-03 & 1.2722646e-05 & -2.5313066e-04 \\
1.2668906e-03 & 1.2340367e-05 & -2.5529401e-04 \\
1.2884378e-03 & 1.1958088e-05 & -2.5745737e-04 \\
1.3099849e-03 & 1.1575809e-05 & -2.5962072e-04 \\
1.3315321e-03 & 1.1193531e-05 & -2.6178408e-04 \\
1.3530793e-03 & 1.0811252e-05 & -2.6394743e-04 \\
1.3746264e-03 & 1.0428973e-05 & -2.6611079e-04 \\
1.3961736e-03 & 1.0046694e-05 & -2.6827414e-04 \\
1.4177208e-03 & 9.6644154e-06 & -2.7043750e-04 \\
1.4392679e-03 & 9.2821366e-06 & -2.7260085e-04 \\
1.4608151e-03 & 8.8998578e-06 & -2.7476421e-04 \\
1.4823623e-03 & 8.5175790e-06 & -2.7692756e-04 \\
1.5039094e-03 & 8.1353002e-06 & -2.7909092e-04 \\
1.5254566e-03 & 7.7530213e-06 & -2.8125427e-04 \\
1.5470038e-03 & 7.3707425e-06 & -2.8341763e-04 \\
1.5685509e-03 & 6.9884637e-06 & -2.8558098e-04 \\
1.5900981e-03 & 6.6061849e-06 & -2.8774434e-04 \\
1.6116452e-03 & 6.2239061e-06 & -2.8990769e-04 \\
1.6331924e-03 & 5.8416273e-06 & -2.9207105e-04 \\
1.6547396e-03 & 5.4593484e-06 & -2.9423440e-04 \\
1.6762867e-03 & 5.0770696e-06 & -2.9639776e-04 \\
1.6978339e-03 & 4.6947908e-06 & -2.9856111e-04 \\
1.7193811e-03 & 4.3125120e-06 & -3.0072447e-04 \\
1.7409282e-03 & 3.9302332e-06 & -3.0288782e-04 \\
1.7624754e-03 & 3.5479544e-06 & -3.0505118e-04 \\
1.7840226e-03 & 3.1656755e-06 & -3.0721453e-04 \\
1.8055697e-03 & 2.7833967e-06 & -3.0937789e-04 \\
1.8271169e-03 & 2.4011179e-06 & -3.1154124e-04 \\
1.8486641e-03 & 2.0188391e-06 & -3.1370460e-04 \\
1.8702112e-03 & 1.6365603e-06 & -3.1586795e-04 \\
1.8917584e-03 & 1.2542815e-06 & -3.1803131e-04 \\
1.9133056e-03 & 8.7200265e-07 & -3.2019466e-04 \\
1.9348527e-03 & 4.8972383e-07 & -3.2235802e-04 \\
1.9563999e-03 & 1.0744502e-07 & -3.2452137e-04 \\
1.9779471e-03 & -2.7483380e-07 & -3.2668473e-04 \\
1.9994942e-03 & -6.5711262e-07 & -3.2884808e-04 \\
2.0210414e-03 & -1.0393914e-06 & -3.3101144e-04 \\
2.0425886e-03 & -1.4216702e-06 & -3.3317479e-04 \\
2.0641357e-03 & -1.8039491e-06 & -3.3533815e-04 \\
2.0856829e-03 & -2.1862279e-06 & -3.3750150e-04 \\
2.1072301e-03 & -2.5685067e-06 & -3.3966486e-04 \\
2.1287772e-03 & -2.9507855e-06 & -3.4182821e-04 \\
2.1503244e-03 & -3.3330643e-06 & -3.4399157e-04 \\
2.1718716e-03 & -3.7153431e-06 & -3.4615492e-04 \\
2.1934187e-03 & -4.0976220e-06 & -3.4831828e-04 \\
2.2149659e-03 & -4.4799008e-06 & -3.5048163e-04 \\
2.2365131e-03 & -4.8621796e-06 & -3.5264499e-04 \\
2.2580602e-03 & -5.2444584e-06 & -3.5480834e-04 \\
2.2796074e-03 & -5.6267372e-06 & -3.5697170e-04 \\
2.3011545e-03 & -6.0090160e-06 & -3.5913505e-04 \\
2.3227017e-03 & -6.3912949e-06 & -3.6129841e-04 \\
2.3442489e-03 & -6.7735737e-06 & -3.6346176e-04 \\
2.3657960e-03 & -7.1558525e-06 & -3.6562512e-04 \\
2.3873432e-03 & -7.5381313e-06 & -3.6778847e-04 \\
2.4088904e-03 & -7.9204101e-06 & -3.6995183e-04
\end{bmatrix}$$

References

[1] Mumenthaler SM, Foo J, Choi NC, Heise N, Leder K, Agus DB, et al. The impact of microenvironmental heterogeneity on the evolution of drug resistance in cancer cells. Cancer Inform. 2015 Jul 15;14(Suppl 4):19–31. doi: 10.4137/CIN.S19338

[2] Awward HK, Aggar ME, Mocktar N, Barsoum M. Intercapillary distance measurement as an indicator of hypoxia in carcinoma of the cervix uteri. Int J Radiat Oncol Biol Phys. 1986 Aug;12(8):1329–33.

[3] Baish JW, Stylianopoulos T, Lanning RM, Kamoun WS, Fukumura D, Munn LL, et al. Scaling rules for diffusive drug delivery in tumor and normal tissues. Proc Natl Acad Sci USA. 2011 Feb 1;108(5):1799–803. doi: 10.1073/pnas.1018154108

[4] Bahnson A, Athanassiou C, Koebler D, Qian L, Shun T, Shields D, et al. Automated measurement of cell motility and proliferation. BMC Cell Biology. 2005 Apr 14;6(19). doi: 10.1186/1471-2121-6-19
